# Supplementary material for: Decoding behavior with minimal and interpretable agent models
Source: PLoS Comput Biol. 2026 Aug 7;22(8):e1014585. doi: 10.1371/journal.pcbi.1014585 (PMC13475988; doi:10.1371/journal.pcbi.1014585)
Supplement: Supplementary file 1 [file pcbi.1014585.s001.pdf]

Supporting Information: "Decoding behavior with minimal and interpretable agent model"

Giorgio Nicoletti[1] and Antonio Celani[1,2]

[1]*Quantitative Life Sciences section, The Abdus Salam International Center for Theoretical Physics (ICTP), Trieste, Italy*
[2]*Department of Oncology, Università degli Studi di Torino, Italy*

A. INFERENCE PROBLEM

We consider an agent that makes decisions in a partially observable environment. The agent takes actions a according to a stochastic policy, and receives observations y from the environment. Each action a belongs to a finite set of elementary actions $\mathcal{A}$, and each observation y to a finite set $\mathcal{Y}$. In principle, the agent's decisions may depend on the full history of previous actions, i.e., the policy is described by $P_t^{\text{agent}}(a_t \,|\, a_0, y_0, \dots, a_{t-1}, y_{t-1})$, where the subscript t is a shorthand notation to denote the conditional dependence on the history up to time t. Given a sequence of T observations – which we do not attempt to model, since they are produced by the external environment –, the probability that the agent has taken a sequence of actions is

$$P^{\text{agent}}_{A_{0:T}|Y_{0:T}}(a_0, \dots, a_T \,|\, y_0, \dots, y_T) = P_0^{\text{agent}}(a_0) P_1^{\text{agent}}(a_1 \,|\, y_0, a_0) \prod_{t=1}^{T-1} P_{t+1}^{\text{agent}}(a_{t+1} \,|\, h_t) \tag{1}$$

where $h_t = (y_0, a_0, \dots, y_t, a_t)$ is the history up to time t.

Then, our inference problem is the following. We seek to infer the finite state controller (FSC) that best describes the agent, where the FSC is defined by:

- a finite set of M internal states $\mathcal{M} = \{M_1, \dots, M_M\}$;
- a policy $\pi(a|m)$, describing the probability of taking an action $a \in \mathcal{A}$ from an internal state $m \in \mathcal{M}$;
- an internal computation $g(m'|m, a, y)$, describing the probability of transitioning to a new internal state $m' \in \mathcal{M}$ from a previous state $m \in \mathcal{M}$ after taking action $a \in \mathcal{A}$ and observing $y \in \mathcal{Y}$;
- an initial distribution over the internal states $\rho(m)$.

In particular, the policy is parametrized as the softmax distribution

$$\pi(a|m) = \frac{\exp\left(\theta^{(\pi)}_{a,m}\right)}{\sum_{a'} \exp\left(\theta^{(\pi)}_{a',m}\right)} \tag{2}$$

where $\theta^{(\pi)} \in \mathbb{R}^{A \times M}$ are the parameters we seek to infer, where $A = |\mathcal{A}|$ is the number of elementary actions. Similarly, the internal computation $g(m'|m, a, y)$ is

$$g(m'|m, a, y) = \frac{\exp\left(\theta^{(g)}_{m',m,a,y}\right)}{\sum_{m''} \exp\left(\theta^{(g)}_{m'',m,a,y}\right)} \tag{3}$$

where $\theta^{(g)} \in \mathbb{R}^{M \times M \times A \times Y}$, with $Y = |\mathcal{Y}|$ the number of possible observations. Finally, the initial internal state of the FSC is specified by

$$\rho(m) = \frac{\exp\left(\theta^{(\rho)}_{m}\right)}{\sum_{m'} \exp\left(\theta^{(\rho)}_{m'}\right)} \tag{4}$$

with $\theta^{(\rho)} \in \mathbb{R}^M$.

As reported in the main text, the probability that the FSC generates a sequence of actions given a sequence of observations of length T can be written as

$$P^{\text{FSC}}_{A_{0:T}|Y_{0:T}} = \sum_{m_{0:T+1}} \rho(m_0) \prod_{t=0}^{T} \pi(a_t|m_t) g(m_{t+1}|m_t, a_t, y_t) \tag{5}$$

which does not depend on the internal states, i.e., $m_{0:T+1}$ can be marginalized out exactly. By definition, the FSC that best models the data is the one that minimizes the expected Kullback-Leibler divergence

$$\mathbb{E}\left[D\left(P^{\text{agent}}_{A_{0:T}|Y_{0:T}}\,||P^{\text{FSC}}_{A_{0:T}|Y_{0:T}}\right)\right] = \mathbb{E}\left[P^{\text{agent}}_{A_{0:T}|Y_{0:T}}\log\frac{P^{\text{agent}}_{A_{0:T}|Y_{0:T}}}{P^{\text{FSC}}_{A_{0:T}|Y_{0:T}}}\right] \tag{6}$$

where the expectation value is taken over the sequences of observations. We remark that both distributions must be understood in terms of their transition probabilities, i.e., of the probability of generating an action at each time t.

In order to infer the FSC, we collect N behavioral trajectories of different durations generated by the agent, which we denote as $\{(\tilde{a}_k, \tilde{y}_k)\}_{k=1}^N$. Each behavioral trajectory is a sequence of actions and observations, which, for ease of notation, we denote with

$$(\tilde{a}_k, \tilde{y}_k) = \left(a_0^{(k)}, y_0^{(k)}, \dots, a_{T_k}^{(k)}, y_{T_k}^{(k)}\right) \tag{7}$$

where T_k is the duration of the k-th trajectory. For ease of notation, we will also denote with $h_t = (a_0, y_0, \dots, a_{t-1}, y_{t-1})$ the history up to time t. From these trajectories, we can build the empirical distribution of the data,

$$\hat{P}^{\text{data}}(\tilde{a}, \tilde{y}) = \frac{1}{N}\sum_{k=1}^{N}\mathbb{I}\left[\tilde{y} = \left(y_0^{(k)}, \dots, y_{T_k}^{(k)}\right), \tilde{a} = \left(a_0^{(k)}, \dots, a_{T_k}^{(k)}\right)\right] \tag{8}$$

where $\mathbb{I}$ is the indicator function. Thus, replacing the expectation value in (6) with the empirical distribution, we obtain

$$\begin{aligned}
\mathbb{E}\left[D\left(P^{\text{agent}}_{A_{0:T}|Y_{0:T}}\,||P^{\text{FSC}}_{A_{0:T}|Y_{0:T}}\right)\right] &\approx \sum_{\tilde{a},\tilde{y}}\hat{P}^{\text{data}}(\tilde{a}, \tilde{y})\left[\log\hat{P}^{\text{data}}_{A_{0:T}|Y_{0:T}}(\tilde{a}, \tilde{y}) - \log P^{\text{FSC}}_{A_{0:T}|Y_{0:T}}(\tilde{a}, \tilde{y})\right] \\
&= \frac{1}{N}\sum_{k=1}^{N}\left[\log\hat{P}^{\text{data}}_{A_{0:T}|Y_{0:T}}(\tilde{a}_k|\tilde{y}_k) - \log P^{\text{FSC}}_{A_{0:T}|Y_{0:T}}(\tilde{a}_k|\tilde{y}_k)\right] \\
&= \frac{1}{N}\sum_{k=1}^{N}\log\hat{P}^{\text{data}}_{A_{0:T}|Y_{0:T}}(\tilde{a}_k|\tilde{y}_k) + \mathcal{L}\left(\theta^{(\rho)}, \theta^{(\pi)}, \theta^{(g)}\right)
\end{aligned} \tag{9}$$

where $\mathcal{L}$ is the negative log-likelihood of the FSC. Unrolling over the trajectories, the first term can instead be written as

$$\begin{aligned}
\frac{1}{N}\sum_{k=1}^{N}\log\hat{P}^{\text{data}}_{A_{0:T}|Y_{0:T}}(\tilde{a}_k|\tilde{y}_k) &= \frac{1}{N}\sum_{k=1}^{N}\left[\log\hat{P}^{\text{data}}\left(a_0^{(k)}\right) + \sum_{t=1}^{T_k}\hat{P}^{\text{data}}\left(a_t^{(k)}\,|h_t^{(k)}\right)\right] \\
&\approx H\left(A_{0:T}\,|\,Y_{0:T}\right)
\end{aligned} \tag{10}$$

where $H\left(A_{0:T}\,|\,Y_{0:T}\right)$ is the entropy of the sequence of actions given the observations. We remark here that we often work with a limited number of behavioral trajectories, whereas estimating $H\left(A_{0:T}\,|\,Y_{0:T}\right)$ would require an accurate estimation of the high-dimensional full joint distribution $P^{\text{agent}}(\tilde{a}, \tilde{y})$, which is in practice out of reach. Nevertheless, in some scenarios, such as the parity checker agent (see Figure 2 of the main text), the agent acts deterministically except for the first action, which is stochastic. In this case, (10) simply amounts to the entropy of the distribution of the first action, which can be reliably estimated from the data. For the parity checker agent, which takes either action "A" or "B" at random with equal probability, we thus have $H\left(A_{0:T}\,|\,Y_{0:T}\right) = \log 2$.

Finally, the inference problem requires solving

$$\underset{\text{FSC}}{\operatorname{argmin}}\;\mathbb{E}\left[D\left(P^{\text{agent}}_{A_{0:T}|Y_{0:T}}\,||P^{\text{FSC}}_{A_{0:T}|Y_{0:T}}\right)\right] \tag{11}$$

which amounts to finding the FSC parameters $\theta = \left(\theta^{(\rho)}, \theta^{(\pi)}, \theta^{(g)}\right)$ such that

$$\theta^* = \underset{\theta}{\operatorname{argmin}}\;\mathcal{L}\left(\theta^{(\rho)}, \theta^{(\pi)}, \theta^{(g)}\right) \tag{12}$$

as reported in the main text.

TABLE A. Comparison between Finite State Controllers and Hidden Markov Models for active perception

**Model**	**Internal computation**	**Policy / Emission**	
HMM	$g(m_{t+1}\|m_t)$	$\pi(a_t\|m_t)$	Autonomous dynamics
GLM-HMM	$g(m_{t+1}\|m_t)$	$\pi(a_t\|m_t, y_{t-1})$	Previous observation modulates emissions
IO-HMM	$g(m_{t+1}\|m_t, y_t)$	$\pi(a_t\|m_t, y_{t-1})$	Observations affect both transitions and emissions
Reactive GLM-HMM	$g(m_{t+1}\|m_t)$	$\pi(a_t\|m_t, y_{t-1}, a_{t-1})$	Previous observation and action modulates emissions
FSC	$g(m_{t+1}\|m_t, a_t, y_t)$	$\pi(a_t\|m_t)$	Observations and actions feed back into state transitions

B. FINITE STATE CONTROLLERS AND STATE SPACE MODELS

Comparison with Hidden Markov Models

Finite state controllers may be viewed as a generalized type of state space models (SSMs) [1] with recurrent transitions. Here, we first consider the case where observations are produced by the environment following an action, as in all experimental paradigms presented in the main text. We describe this scenario as "active perception", where the agent actively takes action in order to gather information from the environment. In the literature, these have sometimes been named "information-seeking" actions and belong to the realm of active sensing [2, 3]. This is also a common setting for partially observable Markov decision processes and reinforcement learning in general, where observations can be identified with rewards and are given to the agent after an action [4].

One of the crucial features of FSCs is that transitions between internal states explicitly depend on both actions and observations. Although this recurrence makes the inference problem particularly hard, it allows them to be expressive while retaining interpretability, as we will show in the next sections. First, we briefly compare with other common SSMs (see Table A and Figure Ba-d). In a hidden Markov model (HMM), transitions between internal states are autonomous and depend only on the previous state ($g(m_t|m_{t-1})$), while the policy – also known as the emission probability – depends on the current internal state, as in FSCs ($\pi(a_t|m_t)$). Thus, observations do not influence transitions. A possible generalization is HMMs where emissions are parametrized by a generalized linear model (GLM-HMMs), so that the policy explicitly depends on observations [5]. We note that the policy of GLM-HMMs can depend on other covariates, such as the previous action: we name these models "reactive" GLM-HMMs, as they are able to act based on the previous decision, while leaving the transitions between internal states independent. More generally, in input-output hidden Markov models (IO-HMMs), the dependence on observations enters both the emission probability and the internal computation [6]. However, the basic idea of these state space models is that the dynamics of the internal or hidden states is disentangled from that of the emissions, which is instead a key dependency in FSCs.

Moore and Mealy formulations of FSCs and dependence on past observations

In frameworks for describing behavior and decision-making, policies can be formulated in two ways: they can depend only on the internal state – a Moore formulation –, or they can depend on both the internal state and external observations – a Mealy formulation [4, 7]. As formulated in the main text and so far, FSCs implement a Moore architecture where the policy $\pi(a_t|m_t)$ depends only on the current internal state. In contrast, IOHMMs follow a Mealy machine structure with observation-dependent emissions, which, for the case of active perception, gives $\pi(a_t|m_t, y_{t-1})$ to preserve the causal structure. Nevertheless, FSCs achieve expressiveness through recurrent state updates $g(m_{t+1}|m_t, a_t, y_t)$ rather than observation-dependent policies. That is, since the history $h_t = (a_0, y_0, \ldots, a_{t-1}, y_{t-1})$ is encoded in the internal states, the policy of an FSC implicitly depends on both past actions and observations, allowing them to exhibit history-dependent behavior.

More rigorously, we can always choose a structure of internal states that makes the dependence on the previous observation explicit. One could formulate an equivalent Mealy-type FSC by augmenting the internal state space to explicitly include the previous observation, e.g., $m_t = (\tilde{m}_t, \hat{m}_t)$ where

$$g(m_{t+1}|m_t, a_t, y_t) = g(\tilde{m}_{t+1}|m_t, a_t, y_t)\delta(\hat{m}_{t+1}, y_t) \tag{13}$$

so that $\hat{m}_t$ perfectly remembers the previous observation. The policy would then be $\pi(a_t|\tilde{m}_t, y_{t-1})$, making the dependence on recent observations explicit rather than implicit. While both formulations are mathematically equiv-

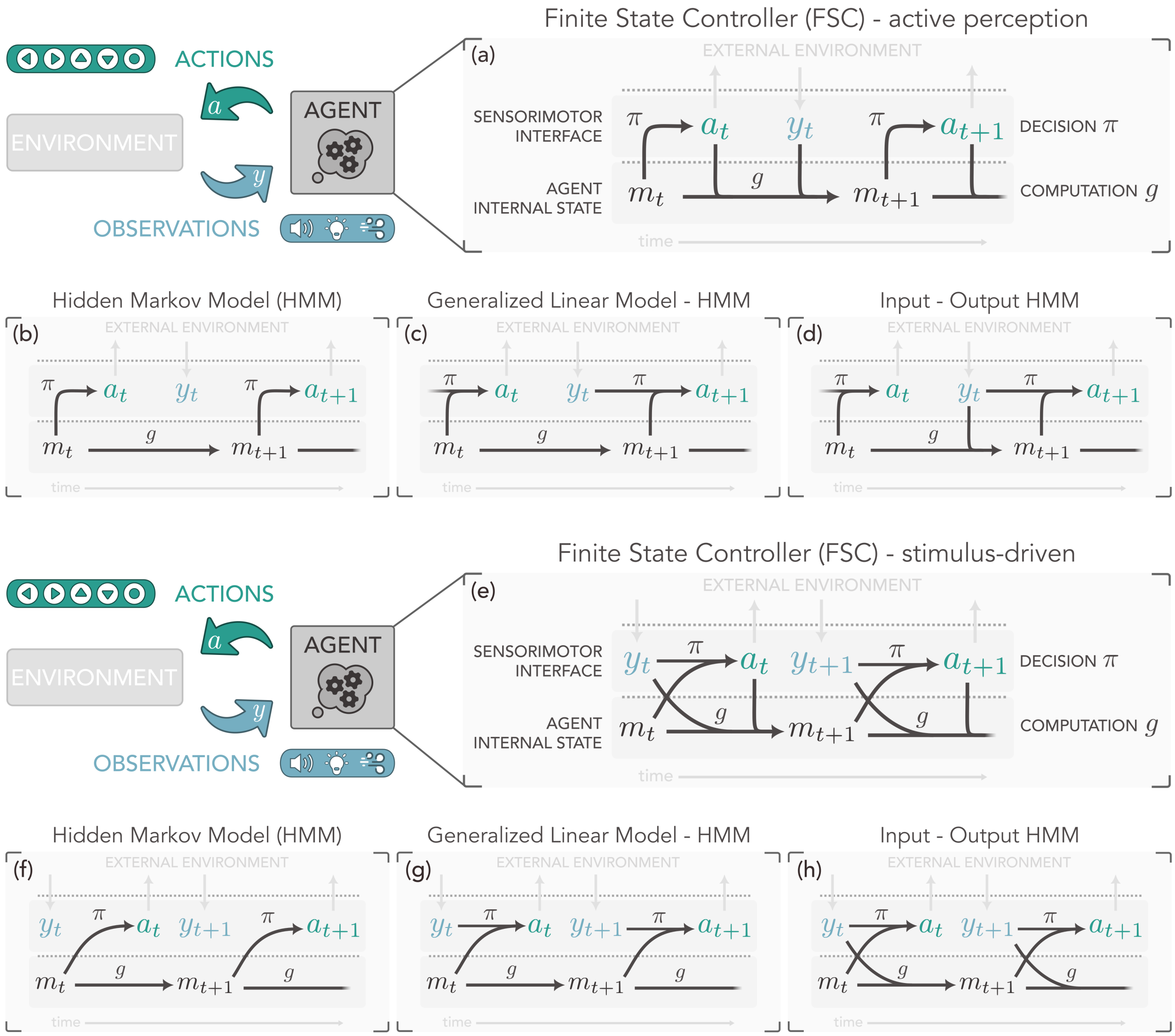

Fig A. Comparison between finite state controllers and other state space models in the case where observations causally follow actions (active perception) and in the case where observations are passively received from the environment and inform actions (stimulus-driven). (a) The FSC model of the agent in the case of active perception, where transitions between internal states are fully recurrent - i.e., they depend on the previous observation, action, and state. (b) In a hidden Markov model (HMM), observations are ignored, and transitions between internal states are autonomous. The policy is known as the emission probability, and solely depends on the current internal state (as in FSCs). (c) In a generalized linear model HMM (GLM-HMM), the transitions between internal states remain autonomous, but the policy depends explicitly on the observation via a generalized linear model [5]. (d) In a general input-output HMM [6], both internal transitions and the policy depend on the observation, but not on the emissions, as in FSCs. (e-h) Same as the previous panel, but for an agent that is stimulus-driven.

alent and equally expressive, the Moore structure offers a more compact state representation, requiring $|\mathcal{M}|$ states instead of $|\mathcal{M}| \times |\mathcal{Y}|$. Furthermore, the Moore formulation provides greater interpretability: each internal state has a well-defined behavioral signature – the actions it produces–, while state transitions capture how the FSC updates its internal representation in response to past observations and actions. This separation between action selection and state update proves particularly useful for understanding the inferred FSCs, as we show in the main text and in later sections.

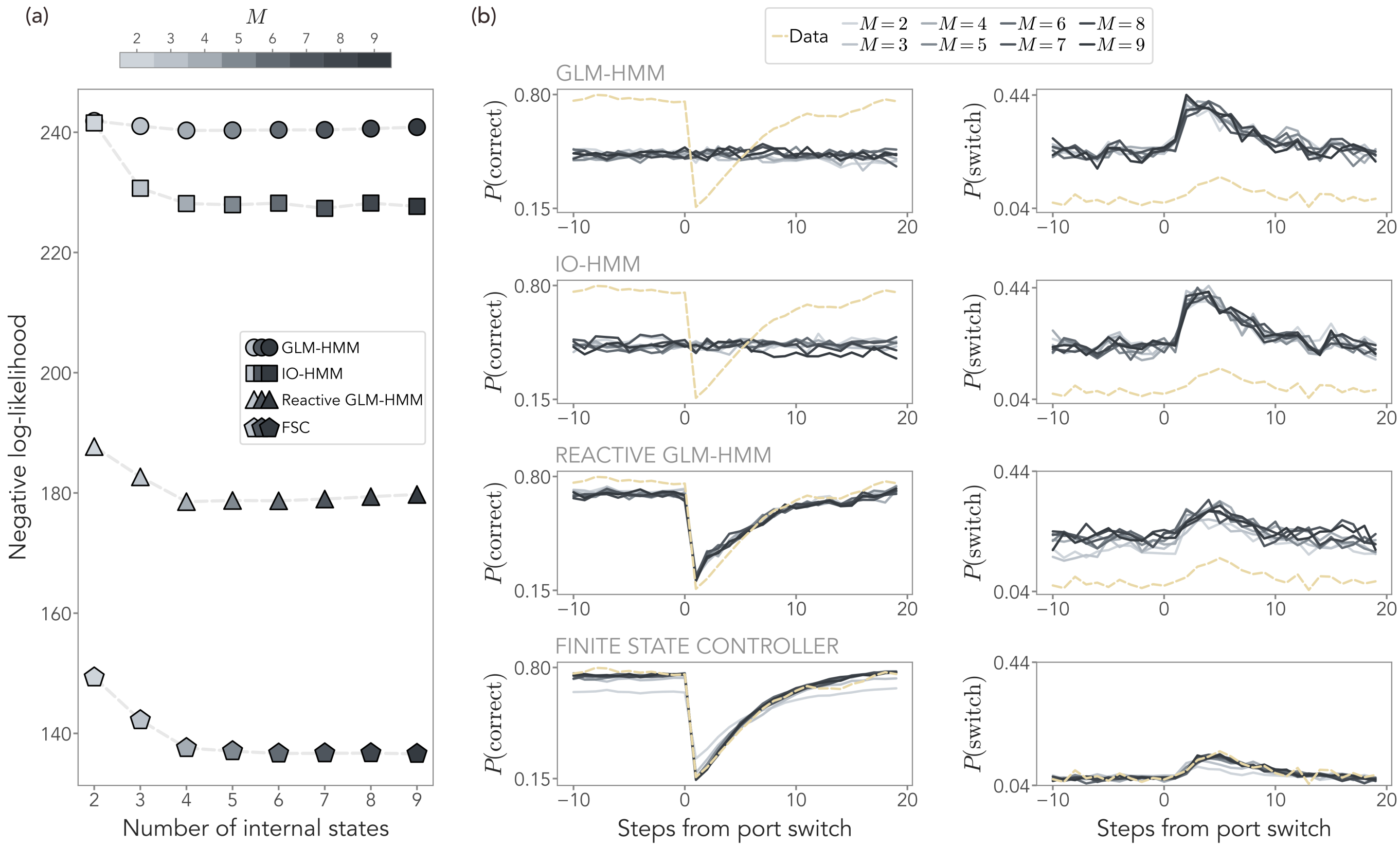

Fig B. Comparison between inferred FSCs and different types of Hidden Markov Models (HMMs) for the mice reversal task. We consider a Generalized Linear Model HMM (GLM-HMM) where the policy depends on the previous observation and the current internal state; an Input-Output HMM (IO-HMM), where both the policy and the internal transitions depend on the previous observation; and a reactive GLM-HMM, where the policy depends on both the previous observation and the previous action. (a) Negative log-likelihood of each inferred model over the validation trajectories for different numbers of internal states. The recurrent structure of FSCs allows them to reproduce the mice behavior more accurately, since their internal states reflect the recent history of past actions and observations. (b) Both GLM-HMM and IO-HMM only take actions based on past observations rather than actions, and thus they cannot reproduce either the probability of the mice selecting the high port, $P(\text{correct})$, nor the probability of switching, $P(\text{switch})$. Reactive GLM-HMMs can fit the former, whereas FSCs with $M \geq 4$ achieve high accuracy on both.

Active perception and stimulus-driven FSCs

Even though we have focused on the case of active perception, both FSCs and our inference method can be formulated to account for scenarios that are instead "stimulus-driven", i.e., where observations are received by the agent before taking actions [8, 9]. As we show in Figure Be, in this case, the natural formulation of an FSC is in the form of a Mealy machine, i.e.,

$$\pi(a_t|m_t, y_t), \quad g(m_{t+1}|m_t, a_t, y_t) \ . \tag{14}$$

We stress that the distinction between active perception and stimulus-driven behaviors strictly depends on the causal structure of the environment, which we do not observe nor model since the likelihood in (5) is completely independent of the environment itself. We also highlight that the dynamics of the environment could be, in principle, extremely complex – e.g., actions taken at a given time may have long-term influences on the observations received by the agent. Furthermore, the behavioral trajectories themselves or the experimental design should determine whether an active perception or a stimulus-driven description is more appropriate. Arguably, one can always describe the decision-making process at a scale where it falls under the active perception framework [2, 10]. For instance, the agent may choose to take an action "perceive" or "sense" to passively receive observations from the environment. Clearly, this may drastically increase the space of possible actions and observations, making a stimulus-driven description more useful in practice. In Figure Bf-h, we compare this FSC with the corresponding HMMs, showing once more how it generalizes their structure by including recurrent transitions and allowing the agent to encode past decisions in its

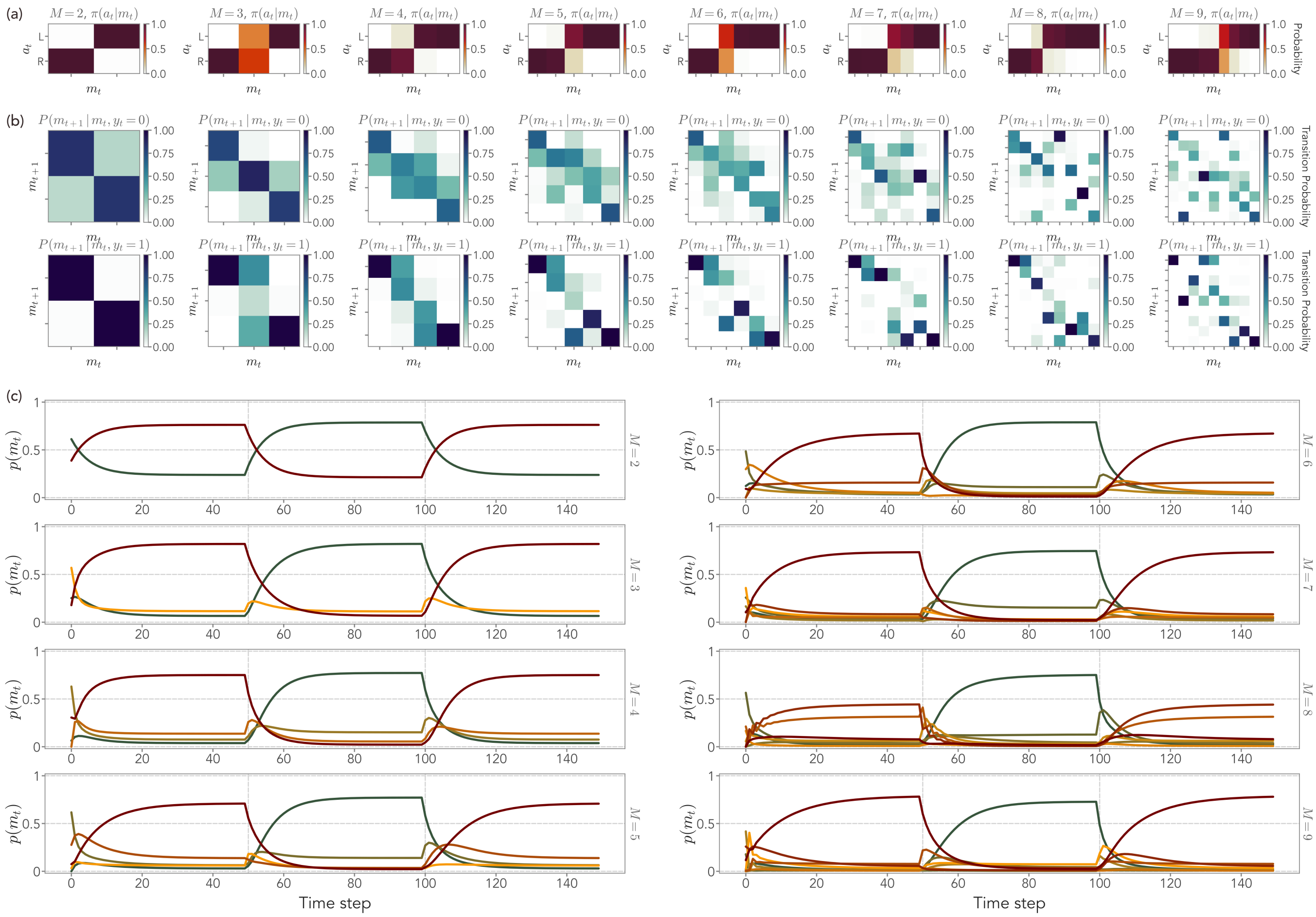

Fig C. Properties of the inferred FSCs for the mice reversal task for different values of M, from $M = 2$ to $M = 9$. From $M = 4$ onward, the negative log-likelihood reaches a plateau (see Figure 5 of the main text). (a) Inferred policies. In all cases, the internal states roughly partition into two groups, each of them taking one of the two available actions (go left or go right). (b) Probability of transitioning from one internal state to the other, if the reward is given ($y = 0$) or not ($y = 1$). As in the main text, a linear structure is clearly visible up to $M = 4$. For $M \geq 5$, the FSC topology remains linear, although several internal states may not be used as the behavior is accurately reconstructed with fewer nodes. (c) Probability of occupying a given internal state for a fixed sequence of ports (see Figure 5g-i of the main text). For $M \geq 3$, switch detectors emerge. For large M, the same internal states may perform the same function – e.g., for $M = 8$ two internal states encode the mice's decisions when the high port has been on the right. As the likelihood suggests, this leads to an equivalent behavior to the case of $M = 4$, which already contains the minimal number of internal states to recapitulate the mice's decision process: two asymmetric switch detectors, and two states for the two ports.

internal states.

Comparison between inferred FSCs and HMMs in the mice reversal task

In Figure B we compare the FSCs inferred from the mice reversal-task data with several types of HMMs. Since, in this case, observations are received after actions, we are in the regime of "active perception". In particular, we consider:

- GLM-HMM with a policy $\pi(a_t|m_t, y_{t-1})$ and internal transitions $g(m_{t+1}|m_t)$;
- IO-HMM with a policy $\pi(a_t|m_t, y_{t-1})$ and internal transitions $g(m_{t+1}|m_t, y_t)$;
- reactive GLM-HMM with a policy $\pi(a_t|m_t, y_{t-1}, a_{t-1})$ and internal transitions $g(m_{t+1}|m_t)$.

In Figure Ba, we show that the validation likelihood is significantly lower for FSCs, and Figure Bb shows that none of the HMMs can recover the the probability of selecting the high reward port $P(\text{correct})$ and the probability of switching

port selection when the high port changes $P(\text{switch})$, both as a function of the time from a switch [11]. Only the reactive version of a GLM-HMM can partially reproduce $P(\text{correct})$. However, the fact that $P(\text{switch})$ cannot be fitted correctly is a consequence of HMMs being unable to encode past actions into their internal state. Indeed, to reproduce the low values of $P(\text{switch})$ at fixed port, with a transient spike around port switches, the agent needs persistence: it must stay committed for many steps when winning, briefly explore when losing, then re-commit. This persistence has to be maintained by the internal state. Thus, while a reactive GLM-HMM can handle the immediate reward-to-action mapping and fit $P(\text{correct})$, it cannot reproduce the mice commitment due to the independence of the internal transitions on the actions. Overall, this shows that the mice's behavior requires non-stationary, input-gated state dynamics, which is provided by FSCs but not HMMs.

C. INFERRED FINITE STATE CONTROLLERS FROM DATA

Linear structure of FSCs for mice reversal task

In Figure C, we show the properties of the inferred FSCs for mice performing a reversal task [11] for different values of M. In all cases, the policy shows that the internal states are partitioned into two groups – the first used to reproduce "go right" action, the second the "go left" – resulting in a linear structure representing the varying degree of commitment of the mice. Importantly, for $M \geq 5$, some states are only occupied very briefly, suggesting that the bulk of the behavior can be reproduced with fewer nodes. As for the $M = 4$ case shown in the main text, central internal states act as switch detectors, i.e., they promote exploratory behavior after the high port switches.

Small FSCs for evidence accumulation data

In Figure D we show the inferred FSCs with $M = 3$ for the dataset of rats performing the evidence accumulation task [12]. In this case, we do not impose any a priori structure. Two classes of equivalent FSCs with $M = 3$ exist. In the first (Figure Da-c), a persistent internal state ( ) takes the "listen" action until the "end" observation is received. Then, it transitions with roughly equal probability to one of two states (and) that take the "go" actions. The second class of FSCs, instead, two states (and) take the "listen" action and switch among themselves depending on the observation. In principle, they could perform computations as in the $M = 4$ case shown in the main text. However, when the "end" is received, only an internal state ( ) is left to take the "go" action, which remains effectively random. In both cases, the FSCs achieve a negative log-likelihood of $\approx \log(2)$, since they can reproduce the "listen" actions perfectly, but are forced to act randomly at the end of the trial.

Nevertheless, this unconstrained inference suggests that, in order to reproduce the data, FSCs with $M \geq 4$ must have a two-layer structure – a computation layer that integrates evidence during the evidence accumulation phase, and a decision layer that is responsible for taking the final decision after the visual cue (the "end" observation) is received. More precisely, the fact that the trials are highly structured – for a trial with T steps, the first $T - 1$ actions are always "listen", while the last action is always the final decision of the rat – causes a likelihood imbalance problem. In fact, since the likelihood is the conditional probability of the sequence of actions given the sequence of observations (Eq. (1), the first $T - 1$ terms outweigh the last one. Thus, imposing the a priori structure constraints the FSCs to reproduce the task structure (taking the "listen" action until the "end" observation is received) and makes it possible for MAPSO to infer the internal transitions that connect the evidence accumulation phase to the final decision. This is equivalent to an inductive bias that forces the FSC to dedicate distinct states to the two phases of the task, effectively sidestepping the imbalance.

Curriculum learning for evidence accumulation data

In Figure E, we show that for $M = 4$ it is possible to avoid the inductive bias described in the previous section and in the main text. To do so, we resort to curriculum learning. As noted above, each trial is naturally split into two parts – the evidence accumulation phase, during which the agent listens, and the decision phase, in which the agent moves and chooses left or right. Thus, we employ curriculum learning to split the inference into two phases as well. In the first, the FSC is trained using short trajectories containing only the last three steps: the last two clicks with the final "end" observation, and the corresponding actions. This allows the FSC to first learn the decision structure in isolation — namely, how to map the final evidence to the correct motor action — without being confounded by the dominant contribution to the likelihood coming from the listening phase. Since these short trajectories contain

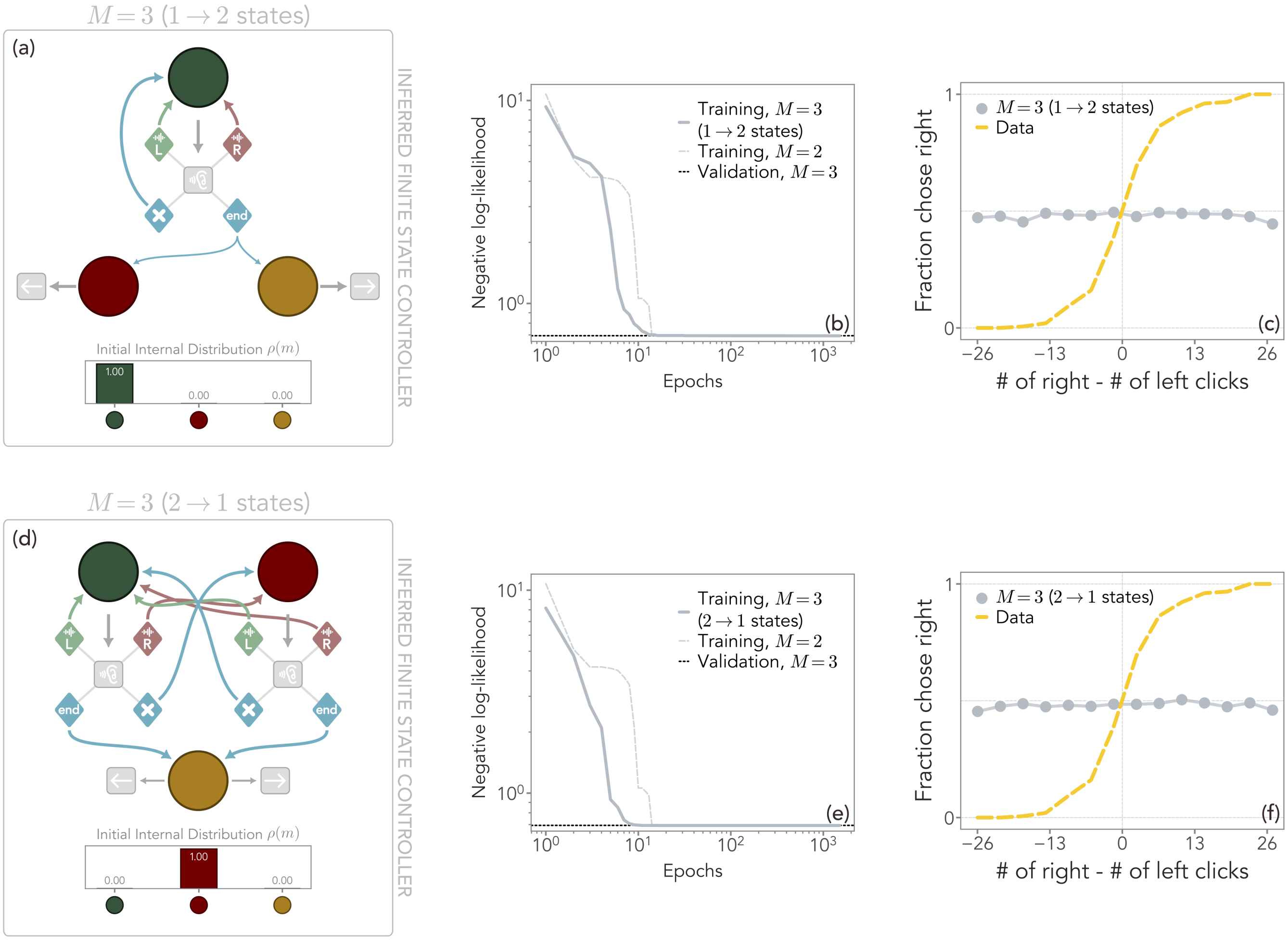

Fig D. Properties of the inferred FSCs with $M = 3$ for rat decision-making during evidence accumulation tasks (see Figure 3 of the main text). Two classes of equivalent FSCs with $M = 3$ exist, and suggest that the behavior can be recapitulated with a two-layer structure for the internal states and for $M \geq 4$. As in the main text, states in the first layer take the "listen" action only, whereas those in the second layer take the "go" actions. (a-c) A first possibility is a two-layer structure with one single node in the first layer ( ) and two in the second (and). The two states in the second layer specialize in taking the two final actions, i.e., going either right or left, after the end observation is received and the trial terminates. In this way, however, no computation can be performed in the remaining state . Thus, in terms of behavior, this topology is fully equivalent to the $M = 2$ one, and indeed it achieves the same negative log-likelihood on the training and validation set (b), as well as acting randomly regardless of the number of left and right observations it received (c). (d-e) The second option is an FSC with two states in the first layer (and) and one in the second ( ). In principle, the nodes in the first layer can now perform computations, as in the $M = 4$ case. However, regardless of the computation, when the end observation is received, the state must switch to and is once again forced to act randomly. In this Figure, no a priori structure was imposed for inference.

only the decision-relevant part of the task, they sidestep the need to reproduce the trivial "listen" actions. As a consequence, the inferred FSC can use all of its internal states to represent the computation that leads to the final choice. Crucially, this first phase spontaneously discovers a layered organization of the internal states, separating computation from action selection, without any architectural constraint being imposed a priori.

In the second phase, we exploit the first by drawing the initial particles' position in MAPSO from a Gaussian distribution with unit variances and means corresponding to the parameters of the FSC inferred in the first phase. The FSC inferred after the second phase of curriculum learning achieves the same negative log-likelihood as the FSC inferred directly using the two-layer constraint, confirming that the two approaches converge to the same solution (Ed). We note that, while curriculum learning allows one to recover the correct FSC without imposing any a priori structure, the two-layer constraint used in the main text remains computationally more efficient and well-motivated by the task design: the separation between a listening phase and a decision phase is an explicit feature of the experimental protocol, not an assumption about the underlying decision computation. Imposing it a priori prevents

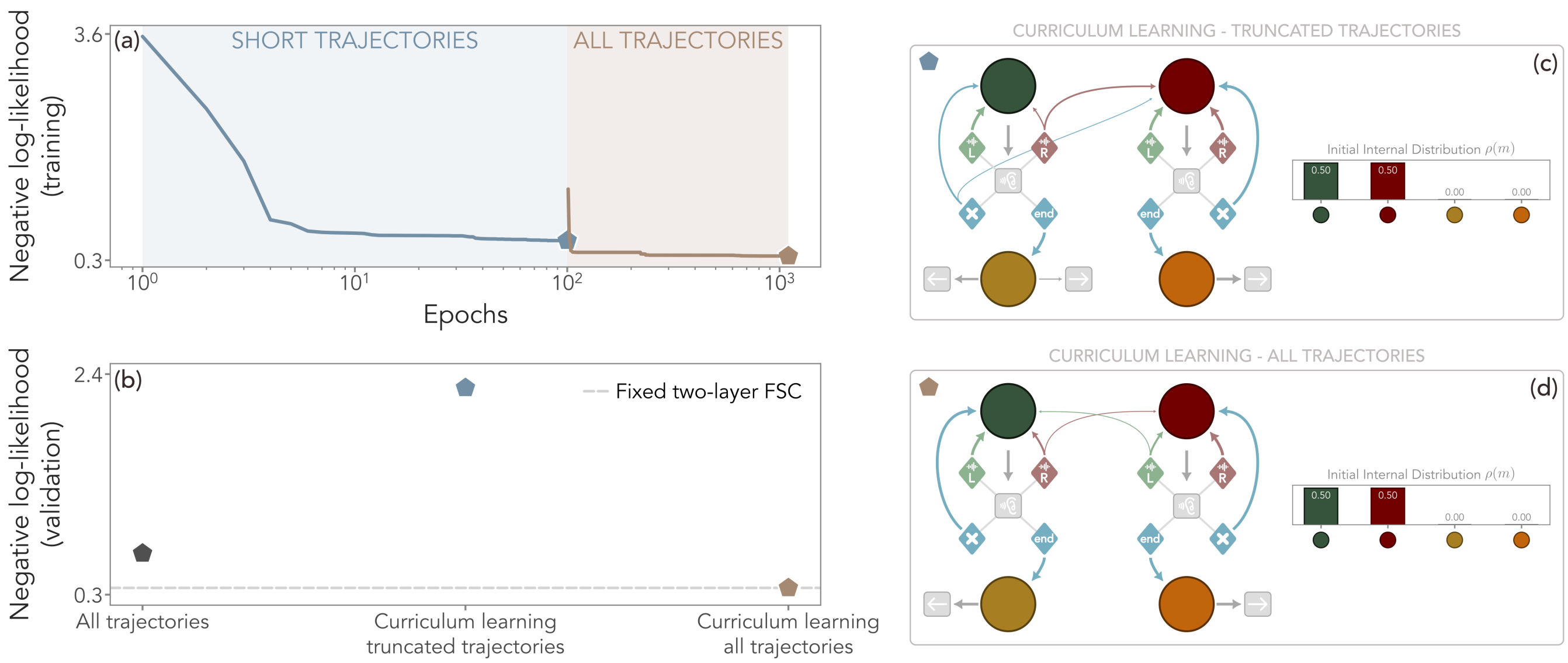

Fig E. Inference of the FSC with $M = 4$ for rat decision-making during evidence accumulation (see Figure 3 of the main text) using curriculum learning. No a priori structure is imposed. (a) Since the task is naturally split into two parts – the evidence accumulation phase, during which the agent listens, and the decision phase – in which the agent moves and chooses left or right, we split inference into two parts as well. In the first part, the FSC is inferred using short trajectories containing only the last three steps: the last two clicks with the final "end" observation, and the corresponding actions. Then, in the second part of curriculum learning, we initialize MAPSO using parameters extracted from a Gaussian distribution with unitary variances and means corresponding to the parameters of the FSC inferred from the first part. (b) The FSC inferred with curriculum learning achieves the same negative log-likelihood on the validation set as the FSC inferred directly from the two-layer structure. (c) The FSC inferred after the first stage of curriculum learning. (d) The FSC inferred after the second stage of curriculum learning, which is identical to that in Figure 3 of the main text.

the optimization from spending resources on reproducing the trivial listening actions. The validity of this constraint can always be verified a posteriori, as FSCs without a two-layer structure achieve a strictly higher negative log-likelihood, and curriculum learning, which imposes no architectural prior, recovers an identical solution. Together, these two checks confirm that the inductive bias introduced by the two-layer structure is benign and does not affect the scientific conclusions drawn from the inferred FSC.

D. INTERPRETING FSCS' INTERNAL COMPUTATIONS

Behaviorally-equivalent FSC and invariance under linear mixing

Before showing how the internal structure of finite state controllers can be explicitly interpreted in terms of behavioral modes and internal computations, we show here that in some cases two different FSCs may sometimes lead to the same behavior. More precisely, two FSCs described by initial distributions ρ and ρ', policies π and π', and internal computations g and g' are behaviorally equivalent if they generate the same conditional probabilities for all histories of actions and observations, i.e., $p(a_{t+1}|h_t) = p'(a_{t+1}|h_t)$ with $h_t = (y_0, a_0, \dots, y_t, a_t)$ as defined above.

In particular, for an FSC with M internal states, we define the observable transition (OT) matrices $(O_{a,y}) \in \mathbb{R}_{\geq 0}^{M\times M}$ as the matrices whose elements $(O_{a,y})_{m'm}$ are equal to the joint transition probabilities

$$O(m', a|m, y) = \pi(a|m)g(m'|m, a, y) \tag{15}$$

which fully describes the FSC, since (15) is nothing but the definition of conditional probability. Then, we seek an invertible matrix $T \in \mathbb{R}_{\geq 0}^{M\times M}$ that leaves the action of the policy on any probability vector invariant, i.e.,

$$\Pi' T = \Pi \tag{16}$$
$$1^T T = 1^T \tag{17}$$

where we denoted with $\Pi \in \mathbb{R}_{\geq 0}^{A\times M}$ the matrix whose entries are the FSC policy. The last condition in (16) implies that T is column-stochastic, so that T represents a convex linear mixing of the internal states. Then, the two FSCs are equivalent if

$$\rho' = T\rho \tag{18}$$
$$O'_{a,y} = T O_{a,y} T^{-1} \quad \forall a, y \tag{19}$$
$$\tag{20}$$

where $p_{h_t} \in \Delta^A$ is the A-dimensional vector whose entries are $p(a_{t+1}|h_t)$, and Δ denotes the simplex. Indeed, if such a T exists, we have that

$$\begin{aligned} p'_{h_t} &= \pi'_{a_{t+1}} O'_{a_t,y_t} \dots O'_{a_1,y_1} O'_{a_0,y_0} \rho' \\ &= \pi_{a_{t+1}} T^{-1} T O_{a_t,y_t} T^{-1} \dots T O_{a_1,y_1} T^{-1} T O_{a_0,y_0} T^{-1} T \rho \\ &= p_{h_t} \end{aligned} \tag{21}$$

so that the two FSCs produce statistically identical behavioral trajectories.

We remark here that, most often, T is trivial. Indeed, if at least two $O_{a,y}$ are full-rank, then T can only be a permutation matrix. Indeed, if T were not a permutation matrix, then some column t_j would have at least two non-zero entries – meaning that state j in one FSC maps to a convex combination of the states of the other FSC, which is incompatible with the similarity on the OT matrices, $TO'_{a,y} = TO_{a,y}$ (see [13] for more details). However, when at least $AY - 1$ OT matrices are not full-rank – i.e., there is at most one OT matrix whose rank is M – then T can be different from a permutation. In the chemotactic agent presented in the main text, this is the case. In Figure F, we show two FSCs that can both be obtained from the inference procedure, both with the same negative log-likelihood – and equal to that of the original chemotactic agent – that are described by

$$\Pi_1 = \begin{pmatrix} 1/2 & 1 & 0 & 1/2 \\ 1/2 & 0 & 1 & 1/2 \end{pmatrix}, \quad \Pi_2 = \begin{pmatrix} 1/2 & 1 & 0 & 0 \\ 1/2 & 0 & 1 & 1 \end{pmatrix} \tag{22}$$

where the first row is run (R), the second tumble (T). The internal computations for the first FSC, the one presented in the main text, are given by

$$G_1(y = \bullet, a = R) = \begin{pmatrix} 0 & 0 & ? & 0 \\ 1 & 0 & ? & 0 \\ 0 & 0 & ? & 0 \\ 0 & 1 & ? & 1 \end{pmatrix}, \quad G_1(y = \times, a = R) = \begin{pmatrix} 1 & 0 & ? & 0 \\ 0 & 0 & ? & 0 \\ 0 & 1 & ? & 1 \\ 0 & 0 & ? & 0 \end{pmatrix} \tag{23}$$

$$G_1(y = \bullet, a = T) = \begin{pmatrix} 0 & ? & 0 & 0 \\ 1 & ? & 1 & 0 \\ 0 & ? & 0 & 0 \\ 0 & ? & 0 & 1 \end{pmatrix}, \quad G_1(y = \times, a = T) = \begin{pmatrix} 1 & ? & 1 & 0 \\ 0 & ? & 0 & 0 \\ 0 & ? & 0 & 1 \\ 0 & ? & 0 & 0 \end{pmatrix} \tag{24}$$

where $G(y, a)$ are the matrices whose elements $G(y, a)_{m'm}$ are equal to the internal computation $g(m'|a, y, m)$, the question marks denote undefined transitions (because the policy does not allow the action from the specific state), and $\{\bullet, \times\}$ represent observing or not observing a chemical, respectively. The second FSC, instead, is described by

$$G_2(y = \bullet, a = R) = \begin{pmatrix} 0 & 0 & ? & ? \\ 1 & 1/2 & ? & ? \\ 0 & 0 & ? & ? \\ 0 & 1/2 & ? & ? \end{pmatrix}, \quad G_2(y = \times, a = R) = \begin{pmatrix} 1 & 0 & ? & ? \\ 0 & 0 & ? & ? \\ 0 & 1 & ? & ? \\ 0 & 0 & ? & ? \end{pmatrix} \tag{25}$$

$$G_2(y = \bullet, a = T) = \begin{pmatrix} 0 & ? & 0 & 0 \\ 1 & ? & 1 & 1/2 \\ 0 & ? & 0 & 0 \\ 0 & ? & 0 & 1/2 \end{pmatrix}, \quad G_2(y = \times, a = T) = \begin{pmatrix} 1 & ? & 1 & 0 \\ 0 & ? & 0 & 0 \\ 0 & ? & 0 & 1 \\ 0 & ? & 0 & 0 \end{pmatrix} \tag{26}$$

and both are initialized in their respective first state.

Consider the matrix

$$T = \begin{pmatrix} 1 & 0 & 0 & 0 \\ 0 & 1 & 0 & 1/2 \\ 0 & 0 & 1 & 0 \\ 0 & 0 & 0 & 1/2 \end{pmatrix} \tag{27}$$

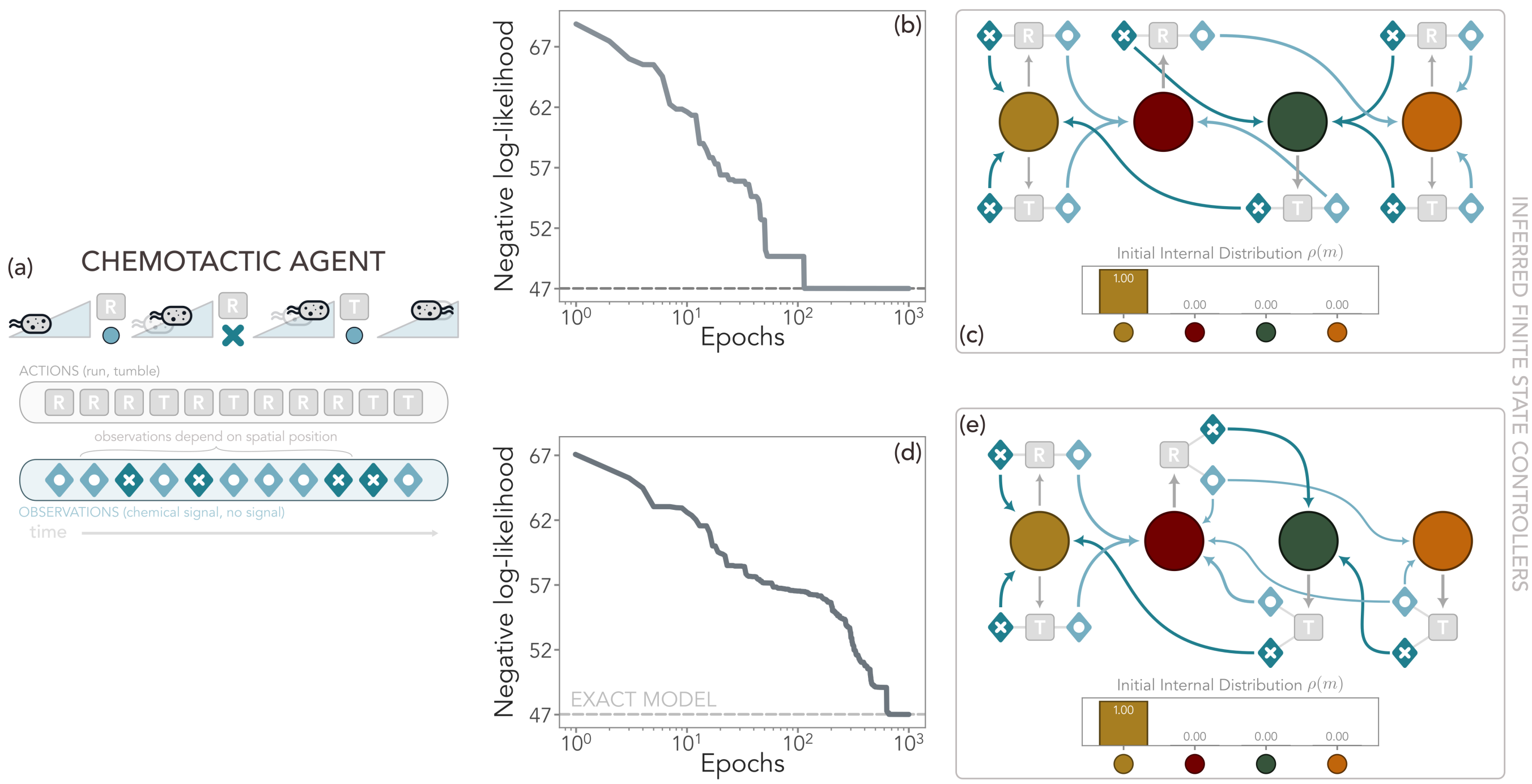

Fig F. Behaviorally-equivalent FSCs inferred for the chemotactic agent introduced in the main text. (a) A chemotactic agent in a $1D$ concentration gradient. At each time, the agent may sense a chemical (observation $\bullet$) or not ($\times$) with a probability that depends on its position. If in the last two steps it received the same observation, the action is chosen at random between tumble (reorienting) and run (move by a fixed amount in the current direction). Instead, if the last two observations have been $\times$ and $\bullet$, respectively, the agent runs, and otherwise it tumbles. (b-c) Negative log-likelihood during training and inferred FSC with $M = 4$ reported in the main text. In this case, the internal states of the FSC represent the four combinations of past observations used by the agent to make decisions: $\sim (y_{t-2}, y_{t-1}) = (\times, \times)$; $\sim (\times, \bullet)$; $\sim (\bullet, \times)$; $\sim (\bullet, \bullet)$. (d-e) A behaviorally-equivalent but different inferred FSC, which reaches the same negative log-likelihood as the previous one and matches that of the original agent. In this case, the stochasticity of the policy when $(y_{t-2}, y_{t-1}) = (\bullet, \bullet)$ is reproduced by equally probable internal transition between the and states, which perfectly reproduce the original diffusive behavior. This equivalence is possible because the internal computation matrices are low-rank.

which is column-stochastic and ensures that

$$\pi_2 T = \pi_1 \tag{28}$$

and satisfies trivially $\rho_2 = T\rho_1$. It is then easy to show that the observable transition matrices,

$$O_1(y=\bullet, a=R) = \begin{pmatrix} 0 & 0 & 0 & 0 \\ 1/2 & 0 & 0 & 0 \\ 0 & 0 & 0 & 0 \\ 0 & 1 & 0 & 1/2 \end{pmatrix}, \quad O_1(y=\times, a=R) = \begin{pmatrix} 1/2 & 0 & 0 & 0 \\ 0 & 0 & 0 & 0 \\ 0 & 1 & 0 & 1/2 \\ 0 & 0 & 0 & 0 \end{pmatrix} \tag{29}$$

$$O_1(y=\bullet, a=T) = \begin{pmatrix} 0 & 0 & 0 & 0 \\ 1/2 & 0 & 1 & 0 \\ 0 & 0 & 0 & 0 \\ 0 & 0 & 0 & 1/2 \end{pmatrix}, \quad O_1(y=\times, a=T) = \begin{pmatrix} 1/2 & 0 & 1 & 0 \\ 0 & 0 & 0 & 0 \\ 0 & 0 & 0 & 1/2 \\ 0 & 0 & 0 & 0 \end{pmatrix} \tag{30}$$

and

$$O_2(y=\bullet, a=R) = \begin{pmatrix} 0 & 0 & 0 & 0 \\ 1/2 & 1/2 & 0 & 0 \\ 0 & 0 & 0 & 0 \\ 0 & 1/2 & 0 & 0 \end{pmatrix}, \quad O_2(y=\times, a=R) = \begin{pmatrix} 1/2 & 0 & 0 & 0 \\ 0 & 0 & 0 & 0 \\ 0 & 1 & 0 & 0 \\ 0 & 0 & 0 & 0 \end{pmatrix} \tag{31}$$

$$O_2(y=\bullet, a=T) = \begin{pmatrix} 0 & 0 & 0 & 0 \\ 1/2 & 0 & 1 & 1/2 \\ 0 & 0 & 0 & 0 \\ 0 & 0 & 0 & 1/2 \end{pmatrix}, \quad O_2(y=\times, a=T) = \begin{pmatrix} 1/2 & 0 & 1 & 0 \\ 0 & 0 & 0 & 0 \\ 0 & 0 & 0 & 1 \\ 0 & 0 & 0 & 0 \end{pmatrix} \tag{32}$$

are all of rank $2 < M = 4$, and satisfy $O_2 = TO_1T^{-1}$ for all a, y, making the two FSC behaviorally equivalent, as expected – since they both reach the negative log-likelihood of the original agent. We also note here that any FSC is properly described in terms of the observable transition matrices alone. For example, in these two cases, some internal computations are not defined because the policy does not allow taking the corresponding action from the corresponding internal state. Because of this, OT matrices are always well-defined.

FSCs with two internal states

As shown in the main text, finite state controllers are expressive enough to reproduce a wide range of behaviors. Importantly, at least in some cases, their finite number of internal states allows for a direct interpretation of their internal computations. Here, we focus on the simplest case of $M = 2$, where we can make general statements. Consider, as above, the $M \times M$ matrix $G(a, y)$ defined by the internal computation $g(m'|m, a, y)$. We denote with $q(a, y)$ the steady state of $G(a, y)$, i.e., the eigenvector of eigenvalue 1 of $G(a, y)$, such that $G(a, y)q(a, y) = q(a, y)$. Similarly, $r(a, y)$ and $l(a, y)$ are the right and left eigenvectors of eigenvalue λ_2 of $G(a, y)$. Then, we have that

$$\begin{aligned} G(a,y) &= q(a,y)1^T + |\lambda_2(a,y)|\, r(a,y)\, l(a,y)^T \\ &= [1 - |\lambda_2(a,y)|]\, q(a,y)1^T + |\lambda_2(a,y)| \left[r(a,y) l(a,y)^T + q(a,y)1^T \right] \\ &= |\lambda_2(a,y)| \mathbb{I} + (1 - |\lambda_2(a,y)|) q(a,y)1^T \end{aligned} \tag{33}$$

where 1 if the vector of all ones and $\mathbb{I}$ is the identity matrix. Therefore, if we now write the probability of occupying an internal state at time t as $p_{\text{mem}}(t) = (p_1(t), p_2(t))$, with $p_2(t) = 1 - p_1(t)$, after taking action a_t and observing y_t this probability evolves as

$$p_1(t+1) = |\lambda_2(a_t, y_t)| p_1(t) + [1 - |\lambda_2(a_t, y_t)|]\, q_1(a_t, y_t) \tag{34}$$

where we have written the components of the stationary state as $q = (q_1, 1 - q_1)$. Therefore, the internal state of the FSC associated with p_1 is computing a discounted sum of the action-observation pairs (a, y), with a discount factor $\lambda_2(a, y) < 1$ that depends on the pair itself. We can interpret this first term in (34) as a forgetting term that manifestly depends on the relaxation timescale of $G(a, y)$. The internal representation of each pair is instead the corresponding stationary state of the dynamics at fixed (a, y), allowing the FSC to encode different pairs in different points of the simplex. Thus, this internal representation effectively depends on how different the stationary states are.

In the case of the computation layer of the FSC with $M = 4$ for the evidence accumulation tasks, for the listen action, we have

$$G(y) = \begin{pmatrix} 1 - \epsilon y \frac{y-1}{2} & \epsilon y \frac{1+y}{2} \\ \epsilon y \frac{y-1}{2} & 1 - \epsilon y \frac{1+y}{2} \end{pmatrix}, \qquad q(y) = \frac{1}{2} \begin{pmatrix} 1 + y \\ 1 - y \end{pmatrix}, \qquad \lambda_2(y) = 1 - \epsilon y^2 \tag{35}$$

where $y \in \{0, \pm 1\}$ for the silence, right, and left observations, and ϵ is the probability of switching from one memory to the other if a contrasting observation is received. Therefore, as in the main text, if we define $\phi(t) = p_1(t) - p_2(t) = 2p_1(t) - 1$, we have that

$$\phi(t+1) = \lambda_2(a_t, y_t)\phi(t) + [1 - \lambda_2(a_t, y_t)]\, [2q_1(a_t, y_t) - 1] \tag{36}$$

which becomes

$$\phi(t+1) = (1 - \epsilon y_t^2)\phi(t) + \epsilon y_t \tag{37}$$

so that the dynamics is fully determined by ϵ. If $\epsilon = 1$, the FSC becomes purely reactive and implements a win-stay-lose-shift dynamics. We also note that, in this simple case where G depends only on y, the two internal states are directly encoding for the left ($y = -1$) or right ($y = 1$) observations, since the silence ($y = 0$) does not change the internal state as expected.

FSCs with an arbitrary number of internal states

Let us define the probability that an FSC with M internal states occupies an internal state at time t as the belief state $b_t \in \Delta^M$, where Δ^M is the M-dimensional simplex. The system receives a discrete input tuple (a_t, y_t) at each time step. As before, we map each unique pair (a, y) to a unique index $k \in \{1, \ldots, K\}$. Then, the belief update is

$$b_{t+1} = G_k b_t \tag{38}$$

where the k index depends on time. We decompose G_k into its stationary and transient components as

$$G_k = \tilde{G}_k + (1-\gamma_k) q_k 1^T \tag{39}$$

where $\gamma_k = |\lambda_2(G_k)|$ is the modulus of the second largest eigenvalue modulus satisfying $|\lambda_2(G_k)| < 1$, and as before q_k is the steady-state distribution of G_k, i.e., $G_k q_k = q_k$. We further decompose the matrix $\tilde{G}_k$ as

$$\tilde{G}_k = H_k + \gamma_k \mathbb{I} \tag{40}$$

so that, since $1^T b_t = 1$, we can write

$$b_{t+1} = \underbrace{\gamma_k b_t}_{\text{Decay}} + \underbrace{(1-\gamma_k) q_k}_{\text{Injection}} + \underbrace{H_k b_t}_{\text{Mixing}} \ . \tag{41}$$

Although in general H_k is at most of rank $M-1$, if $\lambda_2(G_k)$ is real and positive, its rank is $M-2$ since it has two zero eigenvalues – one for the steady-state, and one for the second mode. Indeed, for $M=2$, the mixing term is not present and we recover the case of the previous section.

The first term in (41) represents the memory persistence. The smaller γ_k, the shorter the memory persistence – in the case of $M=2$, this term can be directly interpreted as the exit probability from the internal states. The second term, instead, injects back into the dynamics the steady-state of the current action-observation pair k, with an amplitude that is proportional to the leakage from the current belief b_t, i.e., $1-\gamma_k$. Finally, the last term mixes the different beliefs together, adding or subtracting probability to each internal state depending on the observations and actions. In general, if $\gamma_k \approx 1$ the process is slow-mixing and the FSC tends to preserve memory. If $\gamma_k \approx 0$, the FSC favors new evidence and the process is fast-mixing. Notably, (41) is analogous to a linearized version of a Gated Recurrent Unit (GRU) [14] where the embedding of the k-th action-observation pair is the steady-state of G_k, and without a reset gate (as in light gated recurrent units [15]):

$$b_{t+1} = \gamma_k b_t + (1-\gamma_k)\left[q_k + \frac{1}{1-\gamma_k} H_k b_t\right] \tag{42}$$

where the term in the square bracket is the candidate belief, in analogy with the candidate state of GRUs. This form of the dynamics comes from the fact that the mixing term is originally ungated, so that when $\gamma_k \to 1$ the embedded input q_k is ignored in favor of internal mixing. More detailed interpretations depend on the specific form of G_k inferred from data.

FSC with $M=5$ inferred from evidence accumulation data

We now focus on the FSC with $M=5$ internal states inferred from rats' evidence accumulation data. In particular, we consider the three internal states belonging to the computation layer, since they are responsible for integrating sensory cues – the two remaining states simply project the results of the computation layer into a policy which chooses either right or left. Thus, the components of b_t represent the probability of occupying 🟢, 🟡, 🔴 respectively. Using the notation of the previous sections, we consider for simplicity the perfectly symmetric FSC defined by the transition matrices

$$G(1) = \begin{pmatrix} 1-\xi & 0 & 0 \\ \xi & 1-\eta & 0 \\ 0 & \eta & 1 \end{pmatrix}, \quad G(-1) = \begin{pmatrix} 1 & \eta & 0 \\ 0 & 1-\eta & \xi \\ 0 & 0 & 1-\xi \end{pmatrix} \tag{43}$$

where $G_k = G(y_k)$, since the action is always "listen" in this case, $y = \pm 1$ correspond to hearing right or left, respectively, and $\xi, \eta \in (0,1)$. We ignore the case of $y_t = 0$, since $G(0)$ is simply the identity matrix and no information is present in the observation. Although the inferred FSC is slightly asymmetric due to the noise in the data and the intrinsic asymmetries in the rats' behavior ($p($🟡$|$🟢$, y=1) \approx 0.12$ and $p($🟡$|$🔴$, y=-1) \approx 0.15$, $p($🔴$|$🟡$, y=1) \approx 0.25$ and $p($🟢$|$🟡$, y=-1) \approx 0.29$), it can be numerically checked that this remains an excellent approximation in terms on negative log-likelihood if we use the transition matrices above with average parameters $\xi \approx 0.13$ and $\eta \approx 0.27$. Similarly, the policy can be approximately expressed in terms of these three internal states as

$$\Pi = \begin{pmatrix} 1 & 1/2 & 0 \\ 0 & 1/2 & 1 \end{pmatrix} \tag{44}$$

since, when the "end" observation is received, the states of the computation layer transition to the ones in the second layer, which deterministically choose the action. The first row of Π corresponds to the action "go left", the second to "go right".

Since $\xi < \eta$, for both $G(1)$ and $G(-1)$ we have $\lambda_2 = 1 - \xi$, whereas the stationary states are simply

$$q(1) = \begin{pmatrix} 0 \\ 0 \\ 1 \end{pmatrix}, \quad q(-1) = \begin{pmatrix} 1 \\ 0 \\ 0 \end{pmatrix} \tag{45}$$

which corresponds to the trivial fact that when only $y = 1$ is heard the FSC occupies the state, and if it receives only $y = -1$ instead. In this case, $H(y)$ is a rank-1 matrix, so that

$$G(y) = (1 - \xi)I + \xi q(y)1^T + u(y)v(y)^T \tag{46}$$

where

$$u(1) = \begin{pmatrix} 0 \\ 1 \\ -1 \end{pmatrix} \quad (\text{flow} \rightarrow), \quad u(-1) = \begin{pmatrix} 1 \\ -1 \\ 0 \end{pmatrix} \quad (\text{flow} \rightarrow) \tag{47}$$

represent the flow between the internal states, and

$$v(1) = \begin{pmatrix} \xi \\ \xi - \eta \\ 0 \end{pmatrix}, \quad v(-1) = \begin{pmatrix} 0 \\ \eta - \xi \\ -\xi \end{pmatrix} \tag{48}$$

are the corresponding amplitudes. To make the FSC dynamics more explicit, we define two scalar state variables, ϕ_t^+ and ϕ_t^-, as the projections:

$$\phi_t^+ := -v(-1)^T b_t \tag{49}$$

$$\phi_t^- := v(1)^T b_t \tag{50}$$

where ϕ_t^+ is a positive accumulator representing evidence favoring state ($y = 1$), and ϕ_t^- a negative accumulator representing evidence favoring state ($y = -1$). The dynamics of the pair (ϕ_t^+, ϕ_t^-) forms a switched linear system with asymmetric lateral inhibition. We distinguish two cases. With a positive input ($y_t = 1$) the positive accumulator is *active* (driven), while the negative accumulator is *passive* (decaying):

$$\phi_t^+ = (1 - \xi)\phi_{t-1}^+ - \eta\phi_{t-1}^- + \xi^2 \tag{51}$$

$$\phi_t^- = (1 - \eta)\phi_{t-1}^- \tag{52}$$

so that the passive unit inhibits the active unit. With a negative input ($y_k = -1$), the roles are switched (the negative accumulator is active, and the positive accumulator is passive):

$$\phi_t^+ = (1 - \eta)\phi_{t-1}^+ \tag{53}$$

$$\phi_t^- = (1 - \xi)\phi_{t-1}^- - \eta\phi_{t-1}^+ + \xi^2 \tag{54}$$

and the inhibition direction flips. Crucially, this is a discrete version of a leaky competing accumulator model [16] (LCA) with:

1. accumulation – the active variable integrates the evidence source with a term ξ^2;

2. forgetting – the active unit leaks at rate ξ, the passive unit at a rate η;

3. switched lateral inhibition – the passive unit inhibits the active unit via the term $-\eta\phi_{t-1}^{\mp}$, but not vice versa.

Thus, when the input switches, the new target accumulator is initially suppressed by the inhibition due to the stored value of the old accumulator, generating a dynamics where previous strong commitments are more persistent, as shown in the main text. We also note that the passive decay $(1 - \eta)$ ensures that the inhibition eventually vanishes, allowing the active unit to reach its steady state in the presence of a fully coherent sequence of observations.

In terms of the internal states of the FSC, the accumulators are given by

$$\phi_t^+ = (\xi - \eta) p_t(\textcolor{orange}{\bullet}) + \xi p_t(\textcolor{red}{\bullet}) \tag{55}$$

$$\phi_t^- = \xi p_t(\textcolor{green}{\bullet}) + (\xi - \eta) p_t(\textcolor{orange}{\bullet}) \tag{56}$$

where $p_t(\cdot)$ are the components of b_t at time t, and depend on all the previous observations $y_{0:t-1}$, but we dropped the conditional dependency for brevity. Thus, we can express the probability of choosing an action over the other at the end of the trial at time T as

$$\begin{aligned} \phi(T+1) &= p(A_{T+1} = \text{go right}|y_{0:T}) - p(A_{T+1} = \text{go left}|y_{0:T}) & (57)\\ &= \begin{pmatrix} -1 & 1 \end{pmatrix} \Pi\, b_T = p_T(\textcolor{red}{\bullet}) + \frac{1}{2} p_T(\textcolor{orange}{\bullet}) - \left(\frac{1}{2} p_T(\textcolor{orange}{\bullet}) + p_T(\textcolor{red}{\bullet}) \right) & (58)\\ &= p_T(\textcolor{red}{\bullet}) - p_T(\textcolor{green}{\bullet}) = \frac{1}{\xi} \left(\phi_T^+ - \phi_T^- \right) & (59) \end{aligned}$$

where $y_T = \text{end}$. Thus, the final decision is taken as a function of the accumulators, which accumulate observations and compete with each other via inhibition.

E. METRIC-ADAPTIVE PARTICLE SWARM OPTIMIZATION (MAPSO)

MAPSO is a global optimization algorithm based on an adaptive version of particle swarm optimization [17]. Suppose that we have a loss function to minimize, $\mathcal{L}(\theta)$, that depends on some D-dimensional parameters $\theta \in \mathbb{R}^D$. In the case described in the main text, $\mathcal{L}$ is the negative log-likelihood, and $\theta = (\theta^{(\pi)}, \theta^{(g)}, \theta^{(\rho)})$ are the parameters specifying the policy, computation, and initial distribution of an FSC. If A is the number of actions, Y the number of observations, and M the number of internal states of the FSC, then $D = MA(1 + MY) + M$. In general, however, MAPSO can be used with any kind of loss function – e.g., we may include constraints on specific observables that we can write as functions of θ or sparsity constraints.

The core idea of MAPSO is to explore the parameter space by evolving the position of N_p particles, each corresponding to a set of parameters θ_μ for $\mu = 1, \dots, N_p$. The particle interacts with its neighbors through a temporal interaction network $A_{\mu\nu}(t)$ that depends on all particles' positions in the parameter space. More in detail, the i-th component of the μ-th particle is updated according to the swarming dynamics

$$\begin{aligned} \theta_{\mu,i}(t+1) &= \theta_{\mu,i}(t) + v_{\mu,i}(t+1) \\ v_{\mu,i}(t+1) &= w(t) v_{\mu,i}(t) + c_{\text{cog}}(t) \left[\theta_{\mu,i}^{(\text{cog})}(t) - \theta_{\mu,i}(t) \right] + c_{\text{soc}}(t) \left[\theta_{\mu,i}^{(\text{soc})}(t) - \theta_{\mu,i}(t) \right] \end{aligned} \tag{60}$$

for $i = 1, \dots, D$ and where:

- $w(t)$ is an adaptive inertial parameter;
- $\theta_\mu^{(\text{cog})}(t)$ is the best parameters set ever saw by the μ-th particle after t steps, i.e., $\theta_\mu^{(\text{cog})}(t) = \text{argmin}_\theta \left\{ \mathcal{L}(\theta_\mu(\tau)) \right\}_{\tau=1,\dots,t}$, also known as the "personal best" of the μ-th particle;
- $c_{\text{cog}}(t)$ is an adaptive coefficient that regulates the strength of the "cognitive" dynamics, which makes the particle move in the direction of its personal best;
- $\theta_\mu^{(\text{soc})}(t)$ is the best parameters set ever saw by the *neighbors* of the μ-th particle after t steps, defined at each time according to the corresponding interaction graph $A_{\mu\nu}$, an it is also known as the "local best" of the μ-th particle;
- $c_{\text{soc}}(t)$ is an adaptive coefficient that regulates the strength of the "social" dynamics, which makes the particle move in the direction of its local best.

Finally, depending on the particles' positions, MAPSO attempts to mutate the local best to improve its location. Although different training schedules are possible, we now focus on the case where the algorithm runs for a fixed number of epochs N_{epochs}, and returns the local best with the lowest loss. We will now outline the main steps of MAPSO, and provide a pseudo-code in Algorithm 1.

Initialization

First, the N_p particles are randomly initialized. In our case, we take each initial parameter to follow an independent Gaussian distribution, i.e., $\theta_{\mu,i}(0) \sim \mathcal{N}(0, \sigma^2)$. Similarly, the velocities of the particles are initialized from a uniform distribution $v_{\mu,i}(0) \sim U(v_{\min}, v_{\max})$. We usually take $\sigma^2 \in [0.5, 1, 2.5]$, depending on the problem, and $v_{\max} = -v_{\min} = 10^{-2}$. In general, however, any initialization is possible in principle and, in our experience, will affect the optimization results.

For each particle, the personal best is initialized by definition to the particle's initial position, $\theta_\mu^{(\mathrm{cog})}(0) = \theta_\mu(0)$. We also set $A_{\mu\nu}(0) = 1$ for all $\mu \neq \nu$ and compute the particle for which the loss is lowest, i.e., $\mu^* = \mathrm{argmin}_\mu \left[\mathcal{L}(\theta_\mu(0))\right]$. Since all particles initially interact with one another, each particle's local best is $\theta_\mu^{(\mathrm{soc})} = \theta_{\mu^*}$. Following Ref. [17], we set $w(0) = 0.9$ and $c_{\mathrm{soc}}(0) = c_{\mathrm{cog}}(0) = 2$.

Personal and local best update

After a swarming dynamics step, described in (60), each particle attempts to update both its personal and its local best. We have

$$\theta_\mu^{(\mathrm{cog})}(t+1) = \underset{\theta}{\mathrm{argmin}} \left\{ \mathcal{L}\left(\theta_\mu(t+1)\right), \mathcal{L}\left(\theta_\mu^{(\mathrm{cog})}(t)\right) \right\} \tag{61}$$

so that the personal best is updated with the current position if it has a lower loss. Similarly, the local best is

$$\theta_\mu^{(\mathrm{soc})}(t+1) = \underset{\theta}{\mathrm{argmin}} \left\{ \left\{ \mathcal{L}\left(\theta_\nu^{(\mathrm{cog})}(t+1)\right) \mid A_{\mu\nu} \neq 0 \right\}_{\nu=1,\dots,N_p}, \mathcal{L}\left(\theta_\mu^{(\mathrm{soc})}(t)\right) \right\} \tag{62}$$

so that it is updated if any of the neighbors of the μ-th particle has found a parameter set with a loss lower than the one of the current local best.

Metric interaction network update

The interaction network changes in time according to the metric distance between the particles. Heuristically, MAPSO starts with a "global phase" all particles interact with one another, then evolves towards a "local metric phase", where each particle interacts only with a finite number of neighbors, before moving back to a global phase. In practice, at each step, we compute a number of neighbors $n_{\mathrm{neigh}}(t)$ as

$$n_{\mathrm{neigh}}(t) = \begin{cases} (N_p - 1) + \left(n_{\mathrm{neigh,min}} - (N_p - 1)\right) \left(\frac{t}{N_{\mathrm{epochs}}}\right)^4 & \text{if} \quad t < \frac{N_{\mathrm{epochs}}}{2} \\ n_{\mathrm{neigh,min}} + \left(N_p - 1 - n_{\mathrm{neigh,min}}\right) \left(\frac{2t - N_{\mathrm{epochs}}}{N_{\mathrm{epochs}}}\right)^4 & \text{if} \quad t < \frac{N_{\mathrm{epochs}}}{2} \end{cases} \tag{63}$$

which guarantees that at the beginning and at the end the number of neighbors is equal to $N_p - 1$, while also allowing for an initial slow decay. We empirically found that similar functional forms for $n_{\mathrm{neigh}}(t)$ do not affect the results, as long as they respect these requests.

The interaction network is then an $n_{\mathrm{neigh}}(t)$-nearest neighbors graph, based upon the Euclidean metric. At each step, after determining $n_{\mathrm{neigh}}(t)$, we compute the distance matrix

$$d_{\mu\nu}(t) = \sum_{i=1}^{D} \sqrt{\left[\theta_{\mu,i}(t) - \theta_{\nu,i}(t)\right]^2} \tag{64}$$

and define the interaction network as

$$A_{\mu\nu}(t) = \mathbb{I}\left(d_{\mu\nu}(t) < d_\mu^{(n_{\mathrm{neigh}})}(t)\right) \tag{65}$$

where $d_\mu^{(n_{\mathrm{neigh}})}(t)$ denotes the $n_{\mathrm{neigh}}(t)$-th smallest values among $\{d_{\mu\nu'}(t) \mid \nu' \neq \mu\}$, and we follow the usual convention in graph theory that $A_{\mu\nu}$ defines the interaction from μ to ν. Since the network is undirected by definition, $A_{\mu\nu} = A_{\nu\mu}$.

Adaptive parameters update

The parameters are updated according to the fuzzy rules introduced in Ref. [17]. In particular, an evolutionary parameter $f(t)$ is introduced, defined as

$$f(t) = \frac{d_{\text{best}}(t) - d_{\text{min}}(t)}{d_{\text{max}}(t) - d_{\text{min}}(t)} \in [0,1] \tag{66}$$

where

$$d_{\text{min}}(t) = \min_{\mu} \frac{1}{N_p - 1} \sum_{\nu=1}^{N_p} d_{\mu\nu}(t), \quad d_{\text{max}}(t) = \max_{\mu} \frac{1}{N_p - 1} \sum_{\nu=1}^{N_p} d_{\mu\nu}(t), \quad d_{\text{best}}(t) = \frac{1}{N_p - 1} \sum_{\nu=1}^{N_p} d_{\mu^*(t)\nu}(t) \tag{67}$$

with $\mu^*(t) = \text{argmin}_{\mu} \theta_{\mu}^{(\text{soc})}(t)$. From $f(t)$, we define a membership values S for four different strategies:

$$S_1(f) = \begin{cases} 0 & \text{if } f \leq 0.4 \text{ or } f > 0.8 \\ 5f - 2 & \text{if } 0.4 < f \leq 0.6 \\ 1 & \text{if } 0.6 < f \leq 0.7 \\ -10f + 8 & \text{if } 0.7 < f \leq 0.8 \end{cases} \tag{68}$$

$$S_2(f) = \begin{cases} 0 & \text{if } f \leq 0.2 \text{ or } f > 0.6 \\ 10f - 2 & \text{if } 0.2 < f \leq 0.3 \\ 1 & \text{if } 0.3 < f \leq 0.4 \\ -5f + 3 & \text{if } 0.4 < f \leq 0.6 \end{cases} \tag{69}$$

$$S_3(f) = \begin{cases} 1 & \text{if } f \leq 0.1 \\ -5f + 1.5 & \text{if } 0.1 < f \leq 0.3 \\ 0 & \text{if } f > 0.3 \end{cases} \tag{70}$$

$$S_4(f) = \begin{cases} 0 & \text{if } f \leq 0.7 \\ 5f - 3.5 & \text{if } 0.7 < f \leq 0.9 \\ 1 & \text{if } f > 0.9 \end{cases} \tag{71}$$

and construct the membership value set $(S_1(f), S_2(f), S_3(f), S_4(f))$. The strategy selection at each iteration follows a fuzzy logic rule-based system. Let $\mathcal{A}(f) = \{i \in \{1,2,3,4\} : S_i(f) \neq 0\}$ denote the set of active strategies (those with non-zero membership values) at evolutionary state f. The strategy $S(t)$ at iteration t is selected according to:

$$S(t) = \begin{cases} \arg\max_{i \in \{1,2,3,4\}} S_i(f) & \text{if } |\mathcal{A}(f)| = 1 \\ \min\{j \in \mathcal{A}(f) : j > S(t-1)\} & \text{if } |\mathcal{A}(f)| = 2 \text{ and exists} \\ \min\{j \in \mathcal{A}(f) : j < S(t-1)\} & \text{if } |\mathcal{A}(f)| = 2 \text{ otherwise} \end{cases} \tag{72}$$

where $S(t-1)$ is the previously selected strategy, and the circular ordering wraps around (i.e., after strategy 4 comes strategy 1).

Then, the parameters are updated according to a small positive number $\delta(t) = 0.05(1 + r(t))$, with $r(t)$ a random number between 0 and 1 extracted at each step. If $f(t)$ is relatively large, the strategy should promote "exploration" at the next epoch, i.e., particles should move towards their personal best rather than the local best. Thus,

$$S(t+1) = 1 \implies c_{\text{cog}}(t+1) = c_{\text{cog}}(t) + \delta, \quad c_{\text{soc}}(t+1) = c_{\text{soc}}(t) - \delta\ . \tag{73}$$

If $f(t)$ is relatively small, the strategy should promote "exploitation", i.e., particles should slightly prefer their personal best:

$$S(t+1) = 2 \implies c_{\text{cog}}(t+1) = c_{\text{cog}}(t) + \frac{\delta}{2}, \quad c_{\text{soc}}(t+1) = c_{\text{soc}}(t) - \frac{\delta}{2}\ . \tag{74}$$

Algorithm 1: Metric Adaptive Particle Swarm Optimization (MAPSO)

```
Input: Loss function L(θ), particles N_p, epochs N_epochs, dimension D
Output: Optimal parameter set θ*
// Initialization
for μ = 1 to N_p do
    θ_{μ,i}(0) ~ N(0, σ^2), v_{μ,i}(0) ~ U(v_min, v_max) for i = 1, ..., D;
    θ_μ^(cog)(0) ← θ_μ(0);
A_{μν}(0) ← 1 for all μ ≠ ν, θ_μ^(soc)(0) ← arg min_ν L(θ_ν(0)) for all μ;
w(0) ← 0.9, c_cog(0) ← 2, c_soc(0) ← 2, S(0) ← 1;
for t = 0 to N_epochs − 1 do
    // Swarming dynamics
    for μ = 1 to N_p, i = 1 to D do
        v_{μ,i}(t+1) ← w(t)v_{μ,i}(t) + c_cog(t)[θ_{μ,i}^(cog)(t) − θ_{μ,i}(t)] + c_soc(t)[θ_{μ,i}^(soc)(t) − θ_{μ,i}(t)];
        θ_{μ,i}(t+1) ← θ_{μ,i}(t) + v_{μ,i}(t+1);
    // Update personal and local bests
    for μ = 1 to N_p do
        if L(θ_μ(t+1)) < L(θ_μ^(cog)(t)) then
            θ_μ^(cog)(t+1) ← θ_μ(t+1);
        ν* ← arg min_{ν:A_{μν}≠0} L(θ_ν^(cog)(t+1));
        if L(θ_{ν*}^(cog)(t+1)) < L(θ_μ^(soc)(t)) then
            θ_μ^(soc)(t+1) ← θ_{ν*}^(cog)(t+1);
    // Update metric interaction network
    Compute n_neigh(t) using quartic schedule (global→local→global) and distance matrix d_{μν};
    A_{μν}(t) ← 𝟙(d_{μν}(t) < d_μ^(n_neigh)(t)) where d_μ^(n_neigh) is n_neigh-th smallest distance;
    // Compute evolutionary state and select strategy
    Compute evolutionary state f(t);
    Compute membership values S_1(f), S_2(f), S_3(f), S_4(f) and select S(t+1) via fuzzy logic;
    // Update adaptive parameters based on strategy S(t+1)
    Update c_cog(t+1), c_soc(t+1) according to strategy S(t+1) ∈ {1, 2, 3, 4};
    Clip: c_cog, c_soc ∈ [3/2, 5/2] and normalize if sum > 4;
    w(t+1) ← 1/(1 + (3/2) exp(−13f(t)/5));
    // Mutation step (if in convergence state S(t+1) = 3)
    if S(t+1) = 3 then
        σ(t+1) ← σ_max − (σ_max − σ_min)(t+1)/N_epochs;
        for μ = 1 to N_p do
            Mutate random component of θ_μ^(soc)(t+1) with strength σ(t+1);
            Accept mutation if loss improves;
return arg min_μ L(θ_μ^(soc)(N_epochs))
```

If $f(t)$ is very small, it indicates that particles are close together and the swarm is converging. The strategy should promote "convergence", slightly increasing both the cognitive and the social coefficients:

$$S(t+1) = 3 \implies c_{\text{cog}}(t+1) = c_{\text{cog}}(t) + \frac{\delta}{2}, \quad c_{\text{soc}}(t+1) = c_{\text{soc}}(t) + \frac{\delta}{2} \ . \tag{75}$$

Finally, if f is extremely large, it means that the swarm has split, and a "jumping-out" strategy should be promoted by increasing the social coefficient:

$$S(t+1) = 4 \implies c_{\text{cog}}(t+1) = c_{\text{cog}}(t) - \delta, \quad c_{\text{soc}}(t+1) = c_{\text{soc}}(t) + \delta \ . \tag{76}$$

To avoid too large or too small coefficients, we also apply two clipping strategies, so that neither coefficient can be larger than 5/2 or smaller than 3/2. Then, if $c_{\text{cog}}(t+1) + c_{\text{soc}}(t+1) > 4$, we renormalize them as

$$c_{\text{cog}}(t+1) = 4\frac{c_{\text{cog}}(t+1)}{c_{\text{cog}}(t+1) + c_{\text{soc}}(t+1)}, \quad c_{\text{soc}}(t+1) = 4\frac{c_{\text{soc}}(t+1)}{c_{\text{cog}}(t+1) + c_{\text{soc}}(t+1)} \ . \tag{77}$$

Besides this strategy-based updates of the social and cognitive coefficients, the inertia parameter is also updated according to $f(t)$:

$$w(t+1) = \frac{1}{1 + 3/2 \exp\left(-13 f(t)/5\right)} \in [0.4, 0.9] \tag{78}$$

so that the inertia decreases when f does.

Local best mutations

In the convergence state, where $f(t)$ was small at the previous epochs and the current strategy is $S(t+1) = 3$, we hybridize the particle swarm dynamics with a mutation step. In particular, we attempt to mutate each local best $\theta_\mu^{(\mathrm{soc})}(t+1)$ along a random direction as

$$\theta_{\mu,i^*}^{(\mathrm{soc,mut})}(t+1) = \theta_{\mu,i^*}^{(\mathrm{soc})}(t+1) + r(t+1)\sigma(t+1) \tag{79}$$

where i^* is a random component between 1 and D, $r(t+1) \sim \mathcal{N}(0,1)$, and

$$\sigma(t) = \sigma_{\max} - (\sigma_{\max} - \sigma_{\min}) \frac{t}{N_{\mathrm{epochs}}} \tag{80}$$

is the mutation strength. We typically take $\sigma_{\max} = 1$ and $\sigma_{\min} = 0.1$, to initially favor large mutations and then reduce their strength. For each particle μ, the local best is updated if and only if

$$\mathcal{L}\left(\theta_\mu^{(\mathrm{soc,mut})}(t+1)\right) < \mathcal{L}\left(\theta_\mu^{(\mathrm{soc})}(t+1)\right) \tag{81}$$

and otherwise it is discarded.

F. COMPARISON BETWEEN MAPSO AND LOCAL OPTIMIZATION ALGORITHMS

Given N trajectories $\{(a_{0:T_k}^{(k)}, y_{0:T_k}^{(k)})\}_{k=1}^N$, our goal is to maximize the average log-likelihood

$$\mathcal{L}(\boldsymbol{\theta}) = \frac{1}{N} \sum_{k=1}^{N} \log P_{A_{0:T}|Y_{0:T}}^{\mathrm{FSC}}(a_{0:T_k}^{(k)} | y_{0:T_k}^{(k)}, \boldsymbol{\theta}), \tag{82}$$

where $P_{A_{0:T}|Y_{0:T}}^{\mathrm{FSC}}$ is defined in Eq. (5) and $\boldsymbol{\theta} = (\theta^{(\rho)}, \theta^{(\pi)}, \theta^{(g)})$ for brevity. Besides MAPSO, several other optimization algorithms can be used. Here, we compare MAPSO to two local algorithms, and show that, in some cases, MAPSO systematically outperforms them.

Expectation-maximization for FSCs

The EM algorithm operates directly on the FSC probability tables $\boldsymbol{\phi} = \{\rho, \pi, g\}$ rather than on the parameters $\boldsymbol{\theta}$ introduced above. It iteratively maximizes a surrogate lower bound $F(\boldsymbol{\phi}|\boldsymbol{\phi}^{(\mathrm{old})}) \le \mathcal{L}(\boldsymbol{\phi})$, which touches $\mathcal{L}$ with equality at $\boldsymbol{\phi} = \boldsymbol{\phi}^{(\mathrm{old})}$, guaranteeing that $\mathcal{L}$ is non-decreasing at each iteration.
E-step. For each trajectory k, compute the forward and backward variables

$$\alpha_{t+1}(m') = \pi(a_{t+1}|m') \sum_m \alpha_t(m)\, g(m'|m, a_t, y_t), \quad \alpha_0(m) = \rho(m)\, \pi(a_0|m), \tag{83}$$

$$\beta_t(m) = \sum_{m'} g(m'|m, a_t, y_t)\, \pi(a_{t+1}|m')\, \beta_{t+1}(m'), \quad \beta_{T_k}(m) = 1. \tag{84}$$

These yield the posterior occupation and transition probabilities

$$\gamma_t^{(k)}(m) = \frac{\alpha_t^{(k)}(m)\, \beta_t^{(k)}(m)}{\sum_j \alpha_t^{(k)}(j)\, \beta_t^{(k)}(j)}, \qquad \xi_t^{(k)}(m, m') = \frac{\alpha_t^{(k)}(m)\, g(m'|m, a_t, y_t)\, \pi(a_{t+1}|m')\, \beta_{t+1}^{(k)}(m')}{\sum_j \alpha_{T_k}^{(k)}(j)}. \tag{85}$$

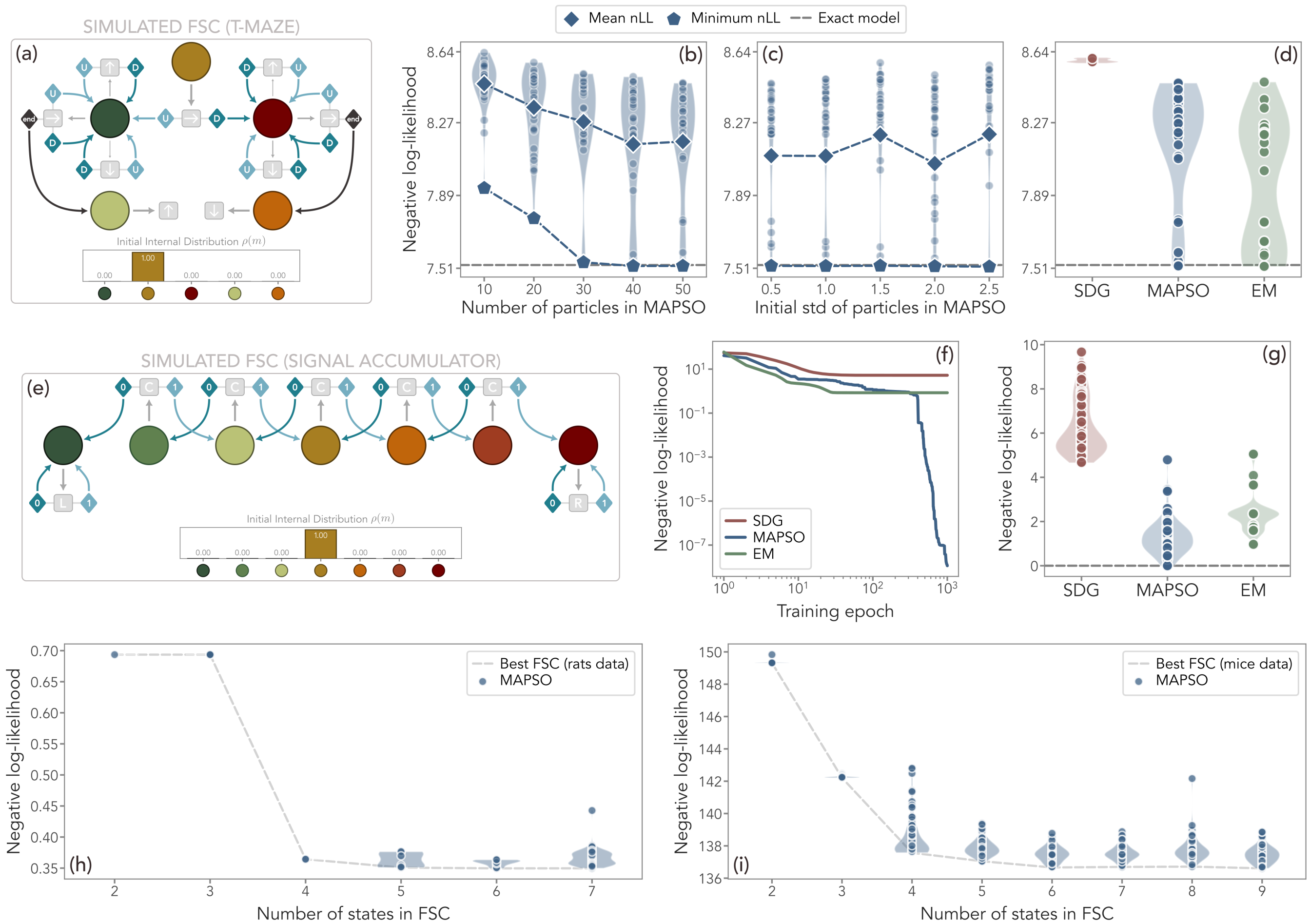

Fig G. Performance of MAPSO with different hyperparameters and comparison with other optimization algorithms. (a) We consider the T-Maze example presented in the main text (Figure 2), where an FSC can perfectly reproduce the agent's behavior. (b) Violin plot of the negative log-likelihood of FSCs inferred with MAPSO across 30 random restarts as a function of the number of particles. With enough particles, MAPSO reliably recovers the exact model. (c) Same as (b), but as a function of the standard deviation (std) of the initial particles' position, which are extracted from a Gaussian distribution with zero mean. Regardless of the position, MAPSO reliably finds the parameters of the exact model. (d) While Stochastic Gradient Descent (SDG) is not able to infer the exact model, Expectation Maximization (EM) achieves a performance similar to that of MAPSO. (e) A deterministic signal accumulator agent described by an FSC. The agent receives two observations (0 and 1), and counts them (action C). After it records three excess counts of 0 (1), it then takes an action "L", left ("R", right). Since the model is deterministic, successful inference corresponds to zero negative log-likelihood. (f) We generate 50 trajectories from the model. Neither SDG nor EM is able to recover the exact model during training, while MAPSO is able to do so. (g) Violin plot of the validation negative log-likelihood across 100 random restarts of the three algorithms. (h-i) Violin plots of the validation negative log-likelihood across random restarts of MAPSO for the rats and mice data.

M-step. Maximizing the surrogate over $\boldsymbol{\phi}$ subject to normalization constraints yields closed-form updates:

$$\rho^{(\mathrm{new})}(m) = \frac{1}{N}\sum_{k=1}^{N} \gamma_0^{(k)}(m), \tag{86}$$

$$\pi^{(\mathrm{new})}(a|m) = \frac{\sum_{k,t} \mathbb{I}(a_t^{(k)} = a)\, \gamma_t^{(k)}(m)}{\sum_{k,t} \gamma_t^{(k)}(m)}, \qquad g^{(\mathrm{new})}(m'|m,a,y) = \frac{\sum_{k,t} \mathbb{I}(a_t^{(k)} = a,\, y_t^{(k)} = y)\, \xi_t^{(k)}(m,m')}{\sum_{k,t} \mathbb{I}(a_t^{(k)} = a,\, y_t^{(k)} = y)\, \gamma_t^{(k)}(m)}. \tag{87}$$

The algorithm iterates E and M steps until convergence of $\mathcal{L}(\boldsymbol{\phi})$.

Stochastic Gradient Descent

Since the likelihood $\mathcal{L}(\boldsymbol{\theta})$ is analytical, it can also be maximized directly via stochastic gradient descent (SGD). At each iteration, a random mini-batch of trajectories is sampled and the gradient of the batch log-likelihood with respect to $\boldsymbol{\theta}$ is computed and used to update the parameters. To ensure convergence, we employ an exponentially decaying learning rate schedule $\eta_s = \eta_0 \, e^{-\lambda s}$, where s is the iteration index and η_0, λ are hyperparameters. The FSC parameters are constrained to the probability simplex at each step by projecting the updates onto the simplex after each gradient step.

Comparison between MAPSO, EM, and SDG

In Figure G, we show the performance of MAPSO with different hyperparameters and compare it with EM and SDG in different scenarios. In all cases, we consider random restarts to mitigate the effect of poor initializations.

In the T-Maze example presented in the main text (Figure 2), MAPSO quickly converges to the exact model that generated the data with enough particles. Notably, the particles' initialization in MAPSO does not influence its performance. In this case, both MAPSO and EM reliably converge to the same solution, while SGD gets stuck on suboptimal minima of the negative log-likelihood.

Then, we consider the specific example of a deterministic signal accumulator. The agent receives two observations (0 or 1) and counts them taking an action "C". After it records three excess counts of 0, it then takes an action "L", and keeps taking it. If the excess count is of 1, it does the same but with an action "R". This model is perfectly representable by an FSC. Further, if the initial distribution ρ is deterministic, the entire model is deterministic as well – meaning that successful inference corresponds to zero negative log-likelihood, regardless of the number of trajectories. We attempt inference with MAPSO, EM, and SDG with very few trajectories ($N = 50$). Neither SDG nor EM is able to recover the exact model during training. On the contrary, MAPSO infers the exact parameters that generated the data. These results, together with those shown in the main text, highlight that MAPSO is particularly well-suited to inference problems where the likelihood landscape is highly non-convex or where the target solution is sharply localized in parameter space.

[1] B. Friedland, *Control system design: an introduction to state-space methods* (Courier Corporation, 2012).
[2] C. E. Schroeder, D. A. Wilson, T. Radman, H. Scharfman, and P. Lakatos, Dynamics of active sensing and perceptual selection, Current Opinion in Neurobiology **20**, 172 (2010).
[3] J. Gottlieb, P.-Y. Oudeyer, M. Lopes, and A. Baranes, Information-seeking, curiosity, and attention: computational and neural mechanisms, Trends in Cognitive Sciences **17**, 585 (2013).
[4] R. S. Sutton, A. G. Barto, *et al.*, *Reinforcement learning: An introduction*, Vol. 1 (MIT press Cambridge, 1998).
[5] Z. C. Ashwood, N. A. Roy, I. R. Stone, I. B. Laboratory, A. E. Urai, A. K. Churchland, A. Pouget, and J. W. Pillow, Mice alternate between discrete strategies during perceptual decision-making, Nature Neuroscience **25**, 201 (2022).
[6] Y. Bengio and P. Frasconi, Input-output hmms for sequence processing, IEEE Transactions on Neural Networks **7**, 1231 (1996).
[7] J. E. Hopcroft, R. Motwani, and J. D. Ullman, *Introduction to Automata Theory, Languages, and Computation*, 3rd ed. (Addison Wesley, 2006).
[8] F. van Ede, A. G. Board, and A. C. Nobre, Goal-directed and stimulus-driven selection of internal representations, Proceedings of the National Academy of Sciences **117**, 24590 (2020).
[9] K. V. B. Verano, E. Panizon, and A. Celani, Olfactory search with finite-state controllers, Proceedings of the National Academy of Sciences **120**, e2304230120 (2023).
[10] S. C.-H. Yang, D. M. Wolpert, and M. Lengyel, Theoretical perspectives on active sensing, Current Opinion in Behavioral Sciences **11**, 100 (2016).
[11] C. C. Beron, S. Q. Neufeld, S. W. Linderman, and B. L. Sabatini, Mice exhibit stochastic and efficient action switching during probabilistic decision making, Proceedings of the National Academy of Sciences **119**, e2113961119 (2022).
[12] T. Z. Luo, T. D. Kim, D. Gupta, A. G. Bondy, C. D. Kopec, V. A. Elliott, B. DePasquale, and C. D. Brody, Transitions in dynamical regime and neural mode during perceptual decisions, Nature **646**, 1156 (2025).
[13] H. Ito, S.-I. Amari, and K. Kobayashi, Identifiability of hidden markov information sources and their minimum degrees of freedom, IEEE Transactions on Information Theory **38**, 324 (1992).
[14] J. Chung, C. Gulcehre, K. Cho, and Y. Bengio, Empirical evaluation of gated recurrent neural networks on sequence modeling, arXiv preprint arXiv:1412.3555 (2014).
[15] M. Ravanelli, P. Brakel, M. Omologo, and Y. Bengio, Light gated recurrent units for speech recognition, IEEE Transactions on Emerging Topics in Computational Intelligence **2**, 92 (2018).
[16] M. Usher and J. L. McClelland, The time course of perceptual choice: the leaky, competing accumulator model., Psychological Review **108**, 550 (2001).
[17] Z.-H. Zhan, J. Zhang, Y. Li, and H. S.-H. Chung, Adaptive particle swarm optimization, IEEE Transactions on Systems, Man, and Cybernetics, Part B (Cybernetics) **39**, 1362 (2009).
